# TCP BBR-n interplay with modern AQM in Wireless-N/AC networks: Quest for the golden pair

**DOI:** 10.1371/journal.pone.0304609

**Published:** 2024-09-23

**Authors:** Muhammad Ahsan, Sajid S. Muhammad

**Affiliations:** 1 Department of Electrical Engineering, National University of Computer & Emerging Sciences, Lahore, Punjab, Pakistan; 2 Department of Software Engineering, University of Management and Technology, Lahore, Punjab, Pakistan; Atlantic Technological University, IRELAND

## Abstract

Effective congestion control on the internet has been a problem since its inception. Transmission Control Protocol (TCP), being the most widely used transport layer protocol tries to mitigate it using a variety of congestion control algorithms. Cubic, Reno, and Bottleneck Bandwidth and Round-trip propagation time (BBR) are the most deployed congestion controls. BBR v2 is leading the congestion control race with its superior performance in terms of better throughput and lower latency. Furthermore, Active Queue Management (AQM) algorithms try to mitigate the congestion control at the network layer through active buffer control to avoid bufferbloat. The most efficient congestion control occurs when TCP and AQM work together. Indeed, it is the TCP-AQM algorithm “*Golden pair*” that can result in the most efficient performance. This paper proposes such a novel pair based on our previously tested and published BBR-n (BBR new) with the most effective of the modern AQMs, that completely gels together to provide lower latency in wireless networks based on Wireless N/AC. Real-time experiments were performed using Flent on our physical testbed with BBR-n and modern AQMs such as Fair Queuing (FQ), Constrained Delay (CoDel), Proportional Integral controller Enhanced (PIE), Common Applications Kept Enhanced (Cake) and Flow Queuing Controlled Delay (FQ_CoDel). Various tests done on our physical testbed helped us identify CAKE as the most optimum AQM that fits with our proposed BBR-n while providing optimum throughput and lower latency in 802.11N/AC-based wireless networks.

## 1 Introduction

**TCP** (Transmission Control Protocol) has been the most deployed transport layer protocol since the last half of the century. It provides end-to-end reliable data transfer between processes running on end systems. Congestion control is also an important feature of it that controls how much a sender can pump into the network making sure that it does not overwhelm the network pipe. As the internet is a packet-switched network, we need buffers in routers to temporarily hold the fast-arriving packets. The size of these buffers is very important as large buffers can cause larger queues and more delays. On the other hand, a relatively small buffer can very easily be filled by a single data burst triggering the congestion control to react incorrectly. This *bufferbloat* [1] problem has been realized and the scientific community has been diligently working on it for its optimum resolution for many decades.

In 1998, the Internet Research Task Force recommended an active queue management technique known as random early detection (RED). Although red was simple and it did handle persistent queues, it was hard to configure. In several cases its performance was poor, and the community was reluctant to use it. The work continued for its improvement and many variants of it were introduced. In 2002, it was proposed by Feng et al. [2] that queue length is not a good way to predict the onset of congestion, but the industry continued with this same old approach.

**Fig 1:**
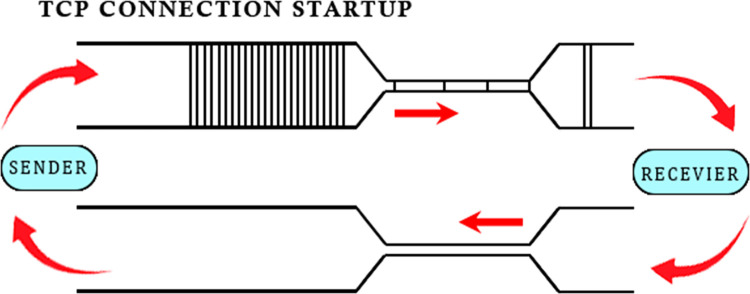
TCP connection startup.

In any statistically multiplexed network, buffers are needed to handle the bursty traffic. **“Fig 1”** shows a TCP connection just after its startup phase. A sender has pumped some packets into the network as per its congestion window (CWND). The packets move through the network until they reach the bottleneck. Here, the packets need to wait in a buffer as the link bandwidth is choked. This forms queues of packets in the available buffers in the link. Now, the size of these buffers is important. It also depends on the link speed, as well as the arrival rate of packets.

Now as packets pile up in the queue, the departure rate of packets increases. Here, it is pertinent to mention that the bandwidth-delay product (BDP) of the link and the congestion window size of the sender are strongly linked with the creation of queues at the bottleneck. If the BDP of the pipe is 20 packets i.e., it needs 20 packets to completely utilize the pipe and on the other hand the sender is sending with a congestion window size of 25 packets, the result would be a standing queue. It is this standing queue that is the root cause of the bufferbloat. It only adds to delays with no gains in the throughput.

**Fig 2:**
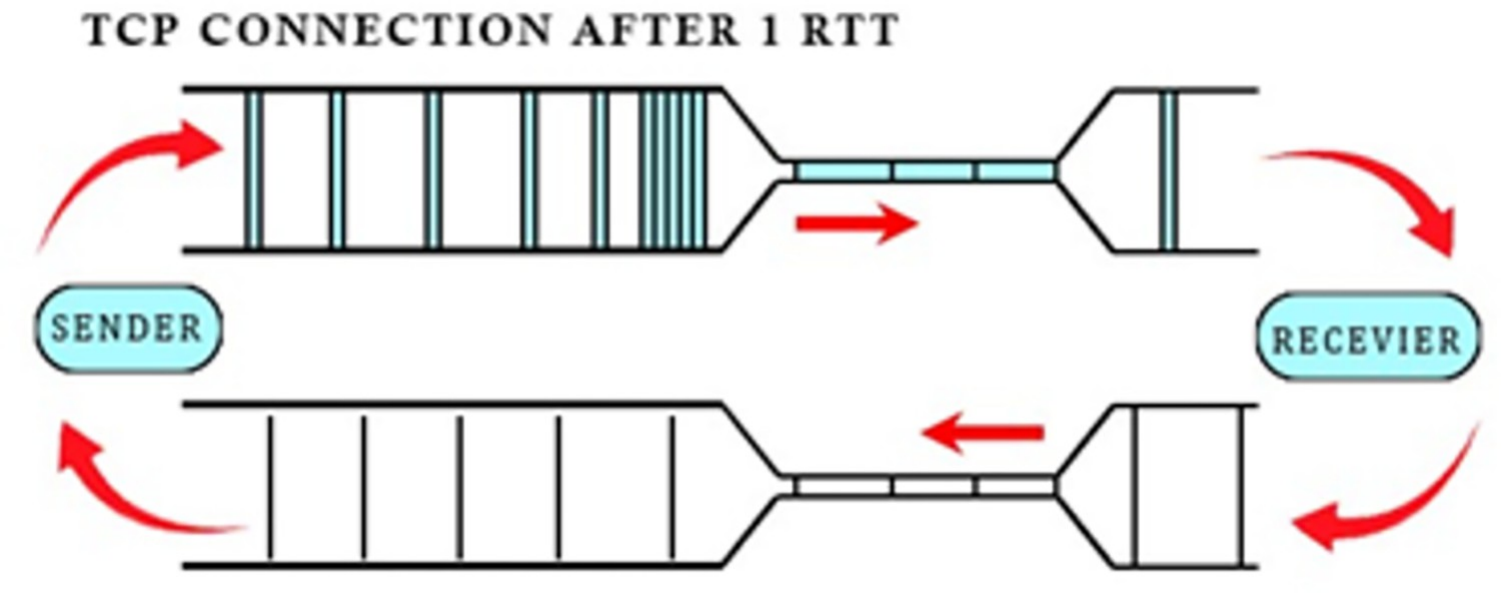
TCP connection after an RTT.

**Fig 3:**
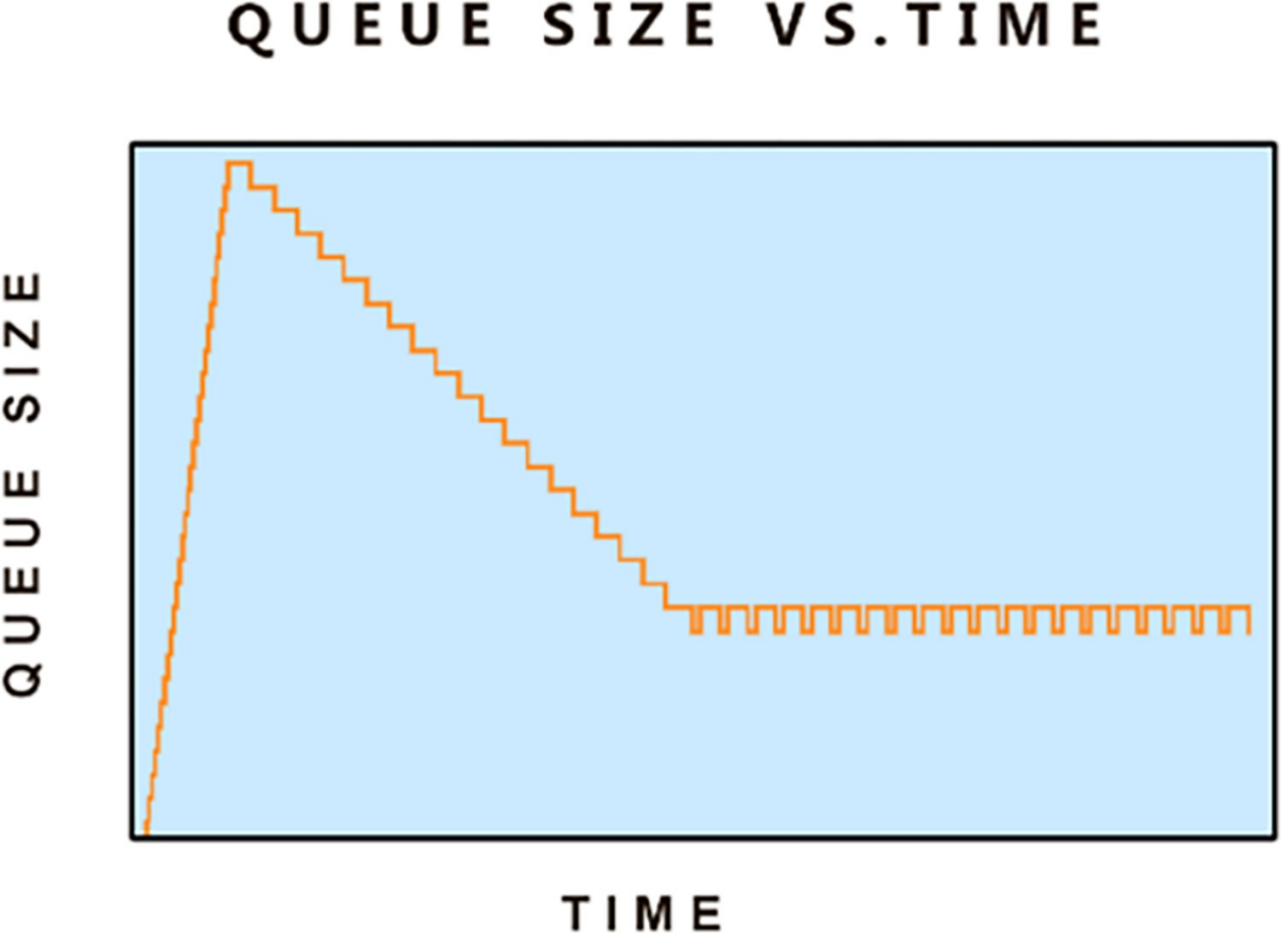
A bad queue.

**“Fig 2”** depicts the scenario after one Round Trip Time (RTT). The bottleneck has spaced out the packets. The receiver is acknowledging the receipt of the data packet with an ACK packet sent on the return path to the sender. ACK stream retains the same spacing on this opposite path. Now the packet arrival rate has become equal to its departure rate and the queue is not increasing. This interesting fact is visible in **“Fig 3”**, which shows the queue size against time.

In its initial phase, the sender is pumping back-to-back packets into the network pipe according to its configured initial congestion window as shown by the gradually rising part of the graph. As the initial CWND is more than the estimated BDP of the pipe, we see that the queue size has started to increase. After an RTT we see the queue size drops, but it does not get to zero and becomes almost constant with minor variations. These variations are due to the time differences between the arrival and departure rates of packets in the buffer. This standing queue is formed as the sender congestion window was assumed 25 packets and the BDP of the pipe was 20 packets. So, at any time there will always be 5 packets in the queue. It is this mismatch between the sender’s CWND and the BDP of the available pipe that creates the standing queue and is the core reason for the bufferbloat.

**Fig 4:**
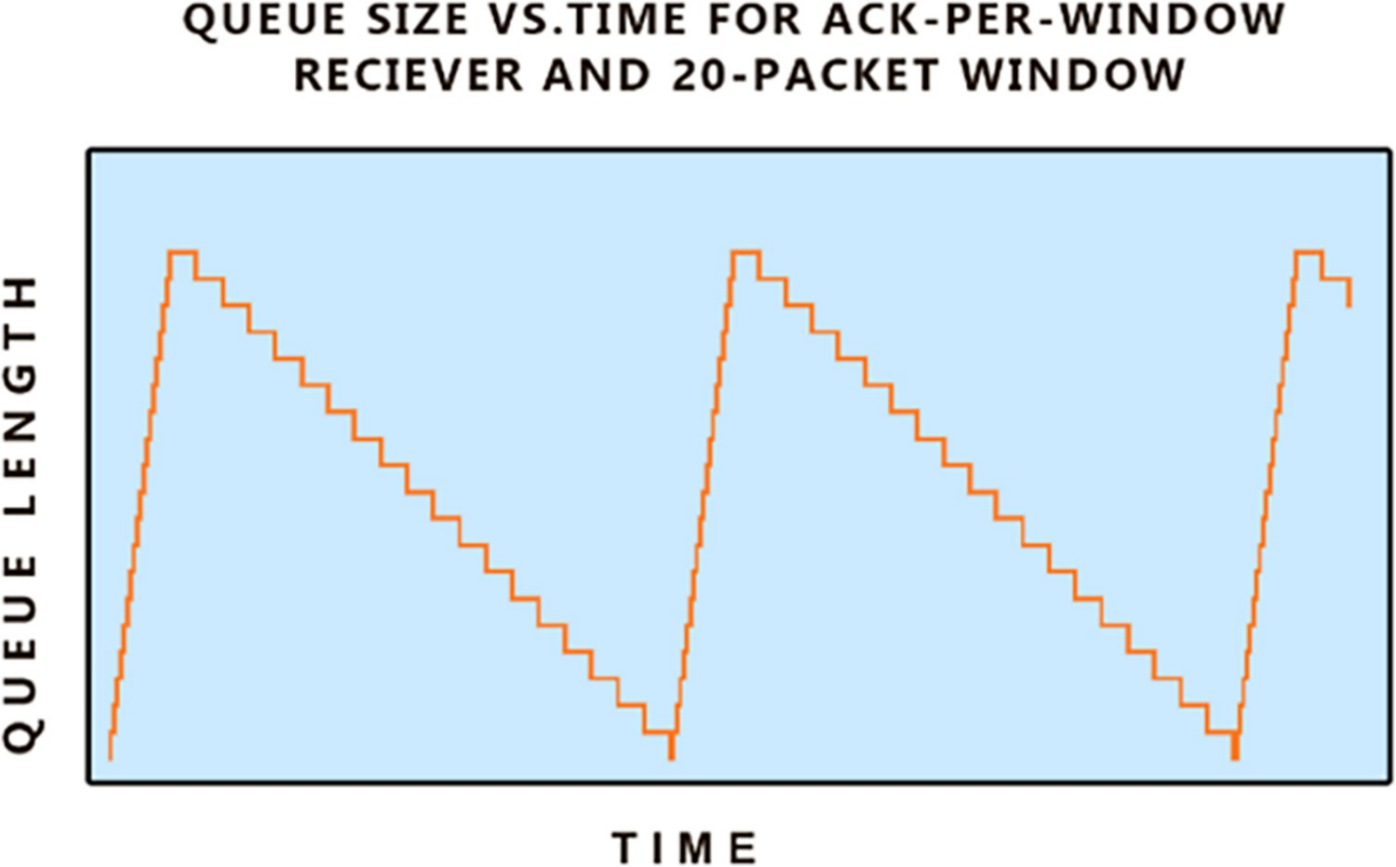
A good queue.

In **“Fig 4”** we see queue occupancy vs. time for a TCP receiver that is using cumulative acknowledgment for the entire window instead of packet per packet base ACK. In **“Fig 4”** and the early phase of **“Fig 3”**, we see that the queues are working optimally and converting bursty traffic arrivals into steady-state departures. This is categorized as a good queue unlike the one shown in **“Fig 3”** which creates delay and is termed as a bad queue.

This issue of bufferbloat [3] was hard to tackle as it was difficult for senders to compute both bottleneck bandwidth and RTT. Thanks to Google’s Bottleneck Bandwidth and Round trip time (BBR) TCP congestion control algorithm that works intelligently in getting this information about the bottleneck bandwidth and RTT from the network periodically using state-of-the-art probing mechanisms and setting up the pacing rate accordingly to keep the pipe just full, but no fuller [4]. Google has released version 3 of BBR (BBR v3) which is expected to reach the Linux mainline code by the end of 2023. We have researched it in a wireless scenario and have proposed our variant of BBR, known as BBR-n (BBR new) [5, 6] that addresses throughput issues of generic BBR when it is deployed with Wi-Fi 4/5 bottlenecks.

Active Queue Management (AQM) techniques are the result of this scientific probe into this crucial issue on the internet. AQM solutions are available at the network layer to manage these buffers in the routers. An ideal congestion control solution is when both the TCP congestion control algorithm (CCA) and the AQM algorithm work together optimally giving the lowest latency. TCP has many CCAs available these days, but the most commonly deployed are the Reno, Cubic, and BBR [7]. Similarly, there have been many flavors of AQMs, but in the last one and a half decade the most common and modern AQMs are Fair Queuing (FQ) [8], Constrained Delay (CoDel) [9], Proportional Integral controller Enhanced (PIE) [10], Common Applications Kept Enhanced (Cake) [11] and Fair Queuing Controlled Delay (FQ_CoDel) [12]. With the evolution of the internet and the tremendous increase in bandwidth requirements, there is a dire need to have robust congestion control and AQM algorithms that can adapt to modern heterogeneous networks. Work on it has been ongoing since the introduction of RED (Random Early Detect) [13] which monitors the average queue size and drops packets based on statistical probabilities. It is better than the conventional tail drop algorithm [14] which leads to TCP global synchronization issues. Evaluation of TCP CCAs, mainly Reno and Cubic with modern AQMs has been done, but there is very little work done on BBR with modern AQMs. With BBR v2 alpha source code already available to the scientific community there is still no work done on evaluating the BBR v2 performance with the modern AQMs such as CAKE and FQ_CoDel. Also, most of the work done is using simulation and wired topology with very little work done on TCP new variants interaction with modern AQMs over wireless physical testbeds.

Finding an optimum TCP-AQM pair is indeed very important for millions of users who are using wireless systems based on Wi-Fi 4/5 to connect to the internet. The issue of bufferbloat can result in significant delays and can be difficult to address for the end user. The home users face very different challenges than the commercial ones. Their links are comparatively slow and highly asymmetric. This can be a real problem, e.g. one member of a home network wireless LAN is pushing a large commit to Git Hub while the other is engaged in real-time combat gaming over the internet involving high stakes. Given the special features of Wi-Fi 4/5 networks, it would indeed make sense to tune the behaviour of the network stack. This is where the need for an optimum TCP-AQM pair comes in. With BBR selected as a TCP congestion control algorithm due to its robust algorithm that provides the best estimate of the available bandwidth, our hunt is to find the second part i.e. an effective AQM that can provide an optimum throughput and lowest latencies in Wi-Fi 4/5 based bottlenecks. Our proposed BBR-n AQM pair is expected to tackle the problems of long queuing delays in WiFi 4/5-based bottlenecks and provide an optimum throughput as well. The latency and throughput tests done over our physical testbed will provide validation. In this paper, we will show through various tests over our physical testbed how TCP BBR-n works with modern AQMs in a wireless 802.11N/AC network scenario. We decided to name the default BBR v2 as *generic BBR* (BBRgen). So, BBRgen will refer to BBR v2 throughout this paper.

The contributions of this paper are as follows: (i) To prove that our proposed BBR-n [6] works better when deployed along CAKE and to some extent with FQ_CoDel schemes than when it is deployed with FQ, PIE, or CoDel algorithms. (ii) The ultimate goal is to find the *Golden Pair* i.e. discovering the AQM algorithm that works best with BBR-n. (iii) The effect of varying TCP Small Queues (TSQ) on BBR-n with their corresponding AQM pairs is also tested via our physical testbed and the results will show that the selection of proper *pacing_shift* [15] is critical in wireless networks. Hence, a need for a TSQ patch [16] along with the proposed BBR-n (BBR new)-AQM pair. (iv) Results of AQM interaction with other popular pure loss-based TCP CCAs such as TCP Cubic and New Reno are also part of the paper, but for the sake of brevity (results of iii and iv) and with Wi-Fi 4 are available at our online data source [17].

The remaining part of our paper is sectioned as follows: Section 2 sheds light on the related works done in this context, along with the security and energy perspective of wireless systems. Section 3 discusses various TCP CCAs, especially the most common, TCP Cubic, BBRgen, and our proposed BBR-n. It provides details of the default parameters available in the five AQMs tested in our paper. Section 4 provides the specification of the physical testbed deployed to generate the results which are presented and discussed in Section 5. Finally, we have Section 6 which provides the conclusion of this paper along with the future directions.

## 2 Related works

The scientific community is actively working on developing state-of-the-art congestion control algorithms for TCP. It has also realized the importance of having an effective AQM algorithm so that both TCP and AQM can join hands with hands in fighting against the well-known issue of congestion on the internet. Afanasyev et al. [18] discuss a wide variety of TCP CCAs, but this study does not take into account the AQMs’ interaction with TCP CCAs. Moreover, since BBR was introduced in 2016 it was not discussed. Plenty of AQM algorithms have been proposed till now, Adams [19] presents a comprehensive survey that discusses various traditional AQMs but misses on PIE and CoDel. Active work on TCP CCAs is in progress and the research community has evaluated various CCAs such as Cubic, Reno, and BBR in different wired [20], wireless [3], and cellular scenarios [21]. Arif et al. in [22] have experimented on various TCP variants by varying the buffer size of the node using network simulator ver. 2 (NS2). Rao et al. [23] analyzed the performance of CoDel and performed a simulation performance analysis with SFQ-CoDel which performs better than generic CoDel. Järvinen and Kojo [24] propose their own modified RED algorithm known as HRED. Comparisons with PIE and CoDel under transient load conditions show that HRED performs better than both PIE and CoDel.

The point we are raising is that individual research work is present both for TCP and AQMs in the scientific world, but research on their optimum combination (TCP-AQM) is relatively less. Raina et al. [25] and Rahman et al. [26] have worked on Compound TCP (CTCP) with old AQMs such as Random Exponential Marking (REM) and Drop-Tail. The work is done using a simulation environment which hides many real-time issues of the physical world. Continuing on the same research track Khademi et al. [27] have tried to explore CoDel and PIE against Adaptive RED (ARED) with only TCP New Reno as the congestion control. TCP Cubic, the most widely used CCA in Linux at that time was not discussed. The paper concludes that ARED’s performance is very near to that of CoDel and PIE. Sun et al. [28] showed that in the TCP-AQM system, the AQM performance suffers when there is two-way traffic due to acknowledgments (ACKs) coming from the receiver side. Sun et al. proposed its solution by assigning priority to ACK packets. Hoiland-Jorgensen et al. [29] used TCP Cubic as the congestion control, with a focus on queuing disciplines available at the bottleneck to provide their experimental evaluation. An early version of BBR was only released in 2016, so it was not evaluated in this work either. In another work done by Anelli et al. [30], an AQM known as FavorQueue was proposed to improve web TCP traffic. It compared itself with the very early algorithms such as DropTail, CHOKe, and TCP New Reno as a congestion control mechanism. Roseberg et al. [31] came up with a unique approach of a man-in-the-middle scenario, in which they proposed a rate management protocol (RMP) that focuses on the Quality of Service (QoS) and fairness. Their proposed RMP protocol controls the rate of all flows in the router and differentiates between aggressive and polite flows. Their approach consists of an RMP ingredient in TCP CCA as well, producing a new variant TCP-RMP as a congestion control algorithm at the nodes and RMP policing the flows at the router. The combination of these two algorithms TCP-RMP and RMP does two works, one was to police aggressive flows being generated by user datagram protocol (UDP) and fast TCP versions, and the other one was by RMP to handle the much polite TCP traffic. On the same grounds, SUN et al. stepped in and proposed an IAPI algorithm [32] and tested it with TCP Reno only. The research works that are the closest to our idea of TCP-AQM pair identification is the Chydzinski and Brachman [33] work done using Cubic and New Reno, but the paper lacks modern AQMs such as PIE, CoDel, and CAKE. **“Table 1”** summarizes it.The reason being their non-availability at that time. The performance metrics they used were only throughout and queue occupancy. We extend this ongoing research to a new dimension by not only bringing into test the latest TCP CCAs such as BBR v2, but also picking five of the most popular AQMs from the last two decades. The five AQMS are chosen as they provide different mechanisms of handling the packets in queues. Each AQM has its typical characteristics that are controlled by their parameters as mentioned in “**Table 2”**. Being available on mainline Linux, all the tested algorithms are available on a wide variety of platforms and have been tested on a wide variety of hardware. In particular, they are part of the open wireless router (OpenWrt) [34] embedded router project, showing that running them on low-powered devices is quite feasible. These AQMs represent algorithms that function well with their default parameters in a wide range of networking environments and heterogeneous topologies. The performance of an AQM at its default parameter is indeed vital for any queuing mechanism’s performance. The difficulties the scientific community faced in configuring RED was one of the major reasons of its limited deployment. We then performed tests of these five AQMs with TCP BBR-n as the main congestion control using a variety of performance metrices such as throughput, queue backlog, TCP latency, RTT fairness latency and web latency were measured for each of the five AQMs.

**Table 1:** Related work in a nutshell.

Work Done By:	TCP-CCA	AQMs (Old/New)	TCP-AQM	Ref.
Afanasyev et al.	Yes	None	None	[18]
Adams	None	Old	None	[19]
Arif et al.	Yes	No	None	[22]
Rao et al.	Yes	Old	None	[23]
Järvinen et al.	Yes	Old	None	[24]
Raina et al.	Yes	Old	yes	[25]
Rahman et al.	Yes	Old	None	[26]
Khademi et al.	Yes	Old	None	[27]
Sun et al.	Yes	Old	None	[28]
Hoiland-Jorgensen et al.	Yes	New	None	[29]
Anelli et al.	Yes	Old	None	[30]
Roseberg et al.	Yes	Old	Yes	[31]
Chydzinski and Brachman	Yes	Old	Yes	[33]
Ahsan and Sajid	Yes	New	Yes	[17]

**Table 2:** Queuing discipline parameters.

Linux Qdisc	Related Parameters	Corresponding Values
**FQ**	limit	10,000 pkts
	quantum	2 * MTU
	bucket	1024
**CoDel**	limit	1000 pkts
	target	5 ms
	interval	100 ms
**PIE**	limit	1000 pkts
	target	15 ms
	tupdate	16 ms
	alpha	2
	beta	20
**CAKE**	bandwidth	300 Mbps
	RTT	100 ms
	triple-isolate	yes
	split/no-split GSO	yes
**FQ_CoDel**	limit	10240 pkts
	target	100 ms
	interval	5 ms
	flow	1024
	quantum	1 eth MTU

AQM, being a viable complementary approach for congestion control, needs more in-depth research, particularly in the wireless domain. Modern AQMs’ interaction with the latest CCA such as BBR v2 along with Cubic and New Reno under one experimental setup is expected to raise greater interest in the scientific community. Our work further extends the above by (a) including the latest TCP CCAs such as BBR variant BBR-n, and loss-based popular CCAs such as Cubic and New Reno. (b) We have included analysis for these TCP CCAs with modern AQMs such as CAKE, FQ_CoDel, and PIE, along with FQ and CoDel. (c) Rigorous tests are performed on a Linux-based physical testbed, providing the complete data set and implementation for reproducing them. We strongly believe that this comprehensive evaluation of the latest TCP CCA particularly BBR-n with modern AQMs will prove vital in understanding the behavior of modern AQMs. Moreover, a physical testbed setup will bring a more realistic picture of TCP-AQM interplay than with simulation setups.

### 2.1 Cybersecurity perspective of TCP-AQM

In the current era of Information Technology with a rapid increase in internet users as well as the availability of large bandwidth cyber-attacks are a known reality. Due to large-scale deployment and application of emerging technologies such as the Internet of Things (IoT) and cloud computing, cyber-attacks against TCP have increased. The denial of service attacks have taken different forms. Distributed as well as the low data rate denial of service attacks known as DDoS and LDDoS are rampant these days over the internet, as well as on the Internet of Things-based networks. As we know TCP congestion control is mainly at the sender side endpoints only, so we need better congestion control at the edge network. Active queue management schemes can play a vital role in mitigating congestion as well as identifying and stopping malicious flows. Bedi et al. [35] propose an AQM here that uses a weighted fair share (wfs) to dynamically adjust edge router buffers according to the congestion along with identifying malicious flows. It keeps track of the state of flows using multiple data structures and cache. The proposed AQM was tested against the AQMS that are almost obsolete these days such as Random early detect (RED) and CHOose and Kill for unresponsive flows (CHOKe).

In general, FQ and AQM technologies are more resistant to simple floods and even DDOS attacks than FIFO. Starting with FQ first, a single DOS (or unresponsive flow) ends up invisible to the overall operation of the queues aside from running the qdisc to its memory limit. FQ_CoDeL then drops robustly (the drop_batch facility drops 64 packets at a time), or CAKE engages the "blue" algorithm which operates on a per-packet basis, usually before it hits the memory limit. Both of these defenses operate independently of whether a packet is ECN-marked or not. AQMs are developing and new flavors are emerging. CAKE which is a result of such a venture. It is logically arranged in several layers of functionality such as shaper, priority queue, flow isolation, AQM, and packet management. Together these layers provide state-of-the-art queuing management as well as security features to identify and drop malicious flows. CAKE implements soft admission control, which makes it robust against starvation attacks relying on strict priority. A major enhancement in CAKE over FQ_CoDeL is the replacement of a plain hash function with an 8-way set-associative hash. Plain hashes are vulnerable to the “birthday problem” [36]. The set-associative hash enables detection and avoidance of collisions. The “count” variable in CAKE now saturates rather than wraps, and in this way handles the overloading conditions optimally. This can be observed with unresponsive and anti-responsive flows with a high packet rate. Further, CAKE can ignore the ECN capability by the packet management layer, if the queue is of out of control length. It will drop instead of marking. This improves robustness against malicious flows that are unresponsive and causing ECN washing.

Here it is pertinent to mention other state-of-the-art approaches to tackle attacks in IP-based networks. Attacks from botnets are rampant these days and their detection is a big challenge. Haq et al. [37] propose two innovative botnet detection approaches using Deep Neural Networks (DNN) methods. Although his work is on IoT devices, the same method may be adopted for wired and wireless IP networks with relevant modifications. Intrusion Detection Systems (IDS) are vital for any enterprise security. To avoid false alarms being generated we need to have systems with a high level of accuracy. Unfortunately, the anomaly-based techniques are not so efficient, and we need to devise a better way. Haq et al. [38] came here with an effective Convolutional Neural Network (CNN) based scheme to detect attacks on edge networks and the accuracy achieved over the explored datasets was above 99%. The same techniques can be utilized for accurate detection of attacks in wireless networks such as those based on WiFi 4/5 bottlenecks. Very recently another botnet detection technique has been proposed by Haq et al. [39] named DBotPM. It is a highly optimized approach that reduces computational overhead by intelligent performance tuning. These latest botnet detection techniques are valid for any real datasets such as those generated in Wi-Fi 4/5 based bottlenecks.

CAKE, which is an evolution of CoDeL and FQ_CoDel is much smarter in detecting malicious flows, and as we will see in the results sections its combination with BBR-n is very good. The innovative methods from Haq et al. can surely come in very handy to further detect any botnets from the real-time datasets gathered.

### 2.2 Energy efficiency perspective of wireless systems

The Wi-Fi Alliance^®^ is striving hard to make a super fast wireless system available for everyone and to connect everything, everywhere, and that too at a cheap cost. The Wi-Fi 4/5 wireless adapters are comparatively cheaper than the faster Wi-Fi 6/7 adapters. All support Multiple-Input Multiple-Output (MIMO). The need for low-cost and energy-efficient wireless systems is the need of the hour. Arfat et al. [40, 41] propose an alternative optimization method that takes into account the effect of nonlinear amplifiers in each transmitter branch, to improve the energy efficiency of massive MIMO. It adapts intelligently to the varying channel conditions on the fly providing optimum energy. Another scientific work by Arfat et al. [41] takes into account the mutual coupling phenomena of transmitting antennas closely placed. Along with achieving energy efficiency in MIMO, the need for an optimum routing mechanism is very important. Anubha et al. [42] propose a routing mechanism based on reward and penalty for the selection of an optimal path that results in less packet drop and is also energy efficient. Kiruthiga et al. [37] focus on Mobile Adhoc Networks (MANETs) node chain connectivity by providing an innovative approach that helps to flow the node chain successively. Together these techniques of enhancing throughput using less costly and energy-efficient wireless devices equipped with robust routing algorithms can indeed prove beneficial for any systems where a TCP-AQM pair has been deployed. Moreover, the discussed approaches provide the scientific community a very good roadmap for further research in improving the wireless system speeds specifically considering energy perspectives and intelligent routing algorithms.

## 3 TCP and AQM algorithms

### 3.1 TCP CCA variants

TCP CCAs have been here for the last 40 years or so. Starting from simple TCP Tahoe, Reno, and Vegas in the early days of the internet when the bandwidths were relatively small. With the evolution of the internet and an increase in the communication links bandwidths several other TCP CCAs [18] such as Compound TCP (CTCP) [43], Data Center TCP (DCTCP) [44], High Speed TCP, Binary Increase Congestion control (BIC), Westwood, Yeah, Veno, Cubic [45] were introduced. Most recently we have BBR v2 (BBRgen). Most of these CCAs were based on detecting congestion based on packet loss alone, with Vegas being the earliest to use delay as a metric for estimating possible congestion. As the internet core bandwidth kept on increasing from Gbps to Tbps more aggressive approaches were needed to utilize the available bandwidth optimally. TCP BIC was introduced in Linux kernel 2.6.8 but was taken over by a more sophisticated and rather less aggressive, but more systematic derivative of BIC, known as TCP Cubic. For many years two of the world’s top operating systems, Linux kept on using TCP Cubic as its default CCA, and Microsoft Windows 7 opted for CTCP, then to NewReno in Windows 8, Cubic in Windows 10, and finally to BBR v2 in Windows 11, 22H2. Linux also includes BBR [15], since Linux kernel 4.9. Now we are at the end of 2023, and TCP Cubic and BBR are the most advanced CCAs. As of now, Google has already started using BBR v3 in its internal WAN traffic and for Google.com public internet traffic [46]. These deployments of BBR and Cubic globally are the main reason that we are researching a BBR variant for Wi-Fi 4/5 and its interplay with modern AQMs.

#### 3.1.1 BBR-n (BBR new)

In our recently published paper [5, 6] we introduced BBR-n, which provides better throughput than generic BBR. It tackles the limitations of generic BBR (BBRgen) by analytically deriving a revised Startup Gain, optimum and aggressive pacing gain strategy, and a suitable transmission/generic segmentation offload (TSO/GSO) chunk for proper frame aggregation that will not starve the Wi-Fi 4/5 of building larger frames. In this paper, which is the continuation of our initial work on BBR. We will be using BBR-n in our ultimate goal of finding the best TCP-AQM pair.

### 3.2. AQM algorithms tested

We selected five of the queueing disciplines (qdiscs) available in the Linux kernel. These five are selected to be tested using their default parameters. To keep testing fair between the five qdiscs we choose to keep their default parameters as it is. Changing default parameters can have effects that can create unfairness among the various tests. How well a qdisc work at its default is indeed a good indicator of its performance. Earlier, some of the queueing algorithms proved difficult to configure and resulted in their limited deployment. RED [14] was the first case.

#### 3.2.1 FQ (Fair Queuing)

FQ is a fairness queueing algorithm [47] that maintains a separate queue for each flow that is currently being serviced by a router. The route then serves these queues in a round-robin fashion. FQ uses TCP pacing and can scale to millions of concurrent flows per disc. It does not tell the traffic sources about the state of the router so that they can limit their sending rate. It has been designed to work with an end-to-end congestion-control algorithm. It uses a hashing mechanism to divide packets into sub-queues. Such hash buckets in the FQ case default to 1024 and each ***bucket*** is assigned a red-black tree for efficient collision sorting. Another important parameter is ***quantum***, which is the number of bytes a flow is allowed to dequeue. As we are sticking to the defaults, the default value of 2 * interface MTU bytes which turns out to be 2 * 1514 = 3028 Bytes is used. The hard ***limit*** on the real queue size remains 10,000 packets.

#### 3.2.2 CoDel (Controlled Delay)

CoDel [48] tries to minimize the delay by noting the sojourn time, which is the actual time a packet experiences in the buffer. Instead of working on queue length, CoDel considers this critical sojourn time. It is this standing queue delay that is measured and compared with the acceptable queue delay ***target***. If the delay remains within this upper bound set by the target, the packets are not dropped. When this delay increases and surpasses a value set by the ***interval***. The defaults for target and interval are 5 ms and 100 ms respectively. The hard ***limit*** on the real queue size for CoDel is 1000 packets. These defaults worked with a large range of internet bandwidths and RTTs, hence the term “no-knobs” for it.

It was developed to address the shortcomings of RED and its variants. CoDel was good at handling bufferbloat problems using its timestamp-based operation. The timestamps are used to detect the minimum queueing delay that the packet faces in a pre-defined interval of 100 ms. When this interval is reached it enters a dropping state. This helps CoDel to tackle typical internet traffic, which is generally bursty. A burst of packets is not dropped if it is cleared from the queue within the specified time. One important feature of CoDel is that it drops packets at the dequeue stage.

#### 3.2.3 PIE (Proportional Integral Controller Enhanced)

PIE [10] is based on a traditional proportional integral controller design. First, it determines the average dequeue rate based on the standing queue. Through which the current queueing delay is computed. Then, periodically this delay is used to calculate the packet drop probability. Finally, when a packet arrives, it gets dropped or marked accordingly. PIE adjusts this probability based on analyzing the delay trend. To control the drop probability growth, it has two statically chosen parameters alpha and beta. Linux kernel 6.x.x uses alpha as 2 and beta to be 20. As already stated earlier in this paper we will stick with the defaults. Just like CoDel, it uses a ***limit*** of 1000 packets. All incoming packets would be dropped if this limit is reached. The default expected ***target*** delay is 15 ms. The frequency at which the drop probability is calculated is governed by ***tupdate***, with a default value of 16 ms in Linux.

#### 3.2.4 CAKE (Common Applications Kept Enhanced)

CAKE [11], is one of the recent queueing disciplines that uses both AQM and FQ. It operates in deficit mode, which is the opposite way of how a token bucket filter (TBF) works. CAKE schedules packets based on time deficits. If there is no deficit, a packet can be scheduled immediately. This mode helps in controlling bursts better than other qdiscs. A variant of deficit round robin (DRR++) scheduler to isolate flows and 8-way set-associative hashing helps combat collisions effectively. Priority queuing is enabled by default using a diffserv3 model. ***Bandwidth*** and round trip time (***RTT***) are its main parameters, we stick with Linux kernel 5.x.x/6.x.x defaults of 100 ms for RTT and unlimited for bandwidth parameters. ***Triple-isolate*** is another critical default parameter for CAKE to prevent any host on the sender/receiver side from monopolizing the link with a large number of flows. ***No-Split-gso*** is a vital parameter available in CAKE and we will see it helps with our proposed BBR-n by not splitting the general segmentation offload (GSO) super-packets, resulting in an increased throughput for Wi-Fi 4/5 for a BBRn-CAKE pair.

#### 3.2.5 FQ_CoDel (flow queueing with controlled delay)

The FQ_CoDel is a hybrid algorithm [49] that combines fair queueing with the CoDel AQM scheme. The authors more generally like it to be called “flow queueing with controlled delay” as the flows that build queues are treated differently from those flows that do not build queues. The classification of incoming packets is done stochastically into different flows and a fair share of bandwidth is provided to all the flows in the queue. CoDel queueing discipline is responsible for managing each of the flows. Reordering within a flow is avoided as CoDel internally uses a FIFO queue. The ***interval*** and ***target*** parameters have the same semantics as CoDel, with default values of 100 ms and 5 ms respectively. The number of bytes used as a deficit in a fair queueing algorithm is controlled by the ***quantum*** parameter and defaults to an ethernet MTU value of 1514 bytes. The number of incoming packets is classified into several flows controlled by a ***flow*** parameter with 1024 as default, a memory limit of 32 MB, and explicit congestion notification (ECN) enabled. The hard ***limit*** in this scheme is set to 10240 packets. **“Table 2”** summarizes the default parameters used for all five AQMs in this paper for the reader’s glance.

## 4 Methodology

**Fig 5:**
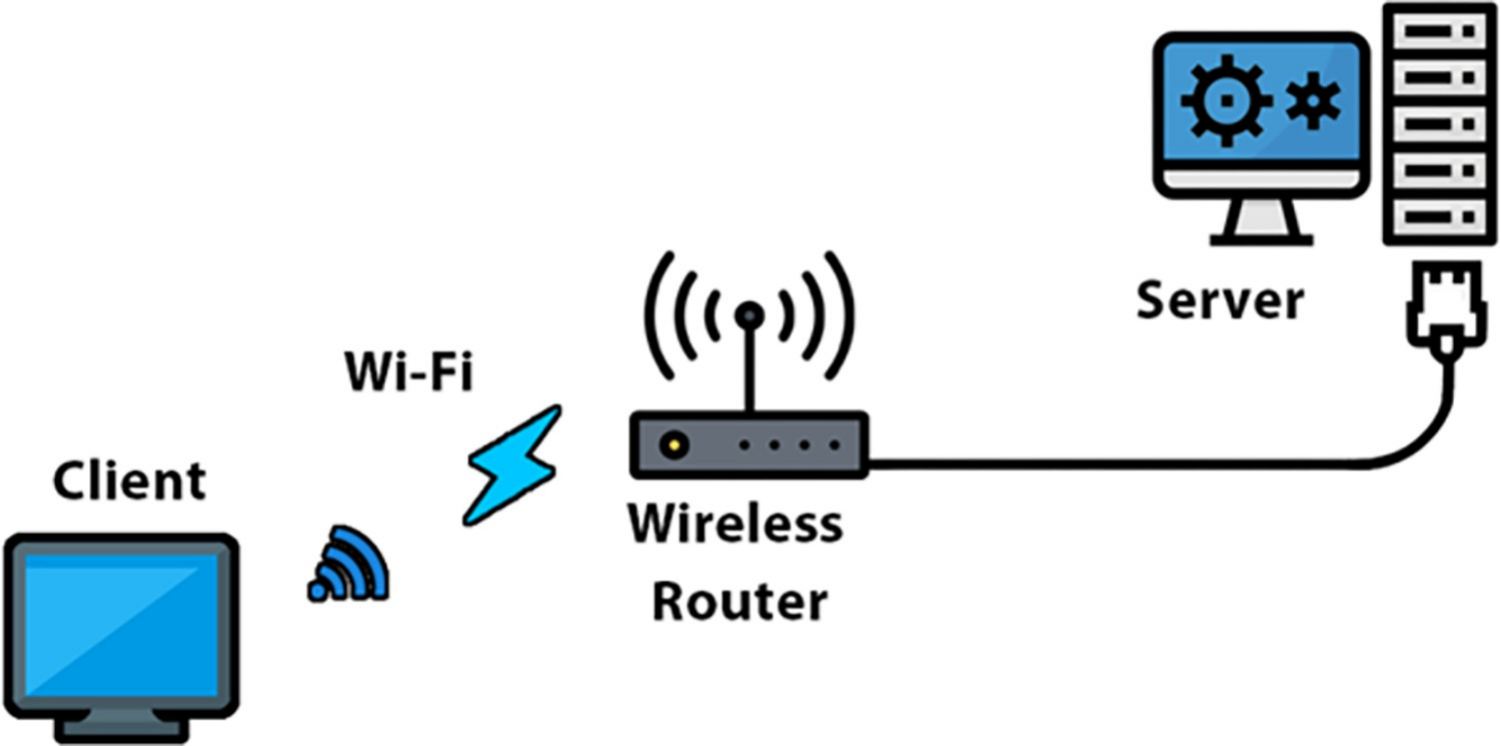
Physical testbed. Shows a Linux-based wireless client connected to a Wi-Fi 4/5router which is wired ethernet to the Linux server. **Image source**: Physical Testbed, “Client, Wireless Router and Server icons” are imported from https://www.pngegg.com/ which is a free hosting site. CC BY 4.0.

This section describes our physical testbed by a schematic representation in **“Fig 5”**. The network topology depicts a ubiquitous form of wireless network found in most homes/offices. It comprises a wireless client, a machine running Linux 5.19.9 kernel, a wireless 802.11n/ac router with OpenWrt [34] support, and servers our ethernet-wired desktop machines running Linux kernel 6.2.0 and 6.1.0. The client machine has a Qualcomm QCA9377 SoC (system on chip) PCIe-based adapter using 256-QAM in a 5 Ghz band. It has Dlink and Realtek USB-based dongles as well for further testing. This testbed will help us analyze the interplay between BBR-n and various AQMs. Each of the selected five AQMs is configured using their default parameters for evaluation purposes to find the best TCP-AQM pair. Our Linux-based client will be focusing on BBR TCP CCA, which is the latest in the domain of congestion controls and has evolved into a superior and robust algorithm since its introduction in 2016 and now in 2023 a much more sophisticated hybrid CCA. Hybrid in the sense that it uses both delay and packet loss as indicators to control the onset of congestion. We used our proposed BBR variant, BBR-n for the conduction of these tests to find the best AQM that suits it. **“Table 2”** provides the default parameter values used for the five AQMs used in this paper and **“Table 3”** provides a detailed testbed specification about the tests involved, performance metrics used, queueing disciplines (Qdiscs) to be evaluated along with TSO/TSQ quantum for bursts and enqueuing.

**Table 3:** Wi-Fi 4/5 testbed specifications.

Related Parameters	Corresponding Values
Clients Kernel Version	5.13.12, 5.15.72 & 5.19.9
Servers Kernel Version	6.2.0-32-generic
	6.1.0-12-amd64
TCP congestion control	BBR v2 (BBRgen), BBR-n, Cubic, Reno. [6]
TCP Small Queues (TSQ) [16]	TSQ (default), 2 TSQ, 4 TSQ, 8TSQ, 16 TSQ
TSO Burst sizes [50]	1,2,4 MSS
Queueing Disciplines	FQ, FQ_Codel, PIE, CAKE, CoDel [11]
	Qualcomm QCA9377 [51]
Wireless chipsets	Dlink 8812BU [52]
	Realtek RTL8821CE [53]
Wireless Driver	rtl88x2bu,ath10k,e1000 [54]
	1/4/8/12 TCP Uploads, Real-time Response under Load Test/Best Effort (RRUL, RRUL_BE),
Tests	
	RTT Fairness, Queue Backlog [55]
Metrics	ICMP Latency (ping RTT)
	TCP Throughput

NetPerf 3 [56] has been set up on both of the Linux servers so that one or both local wired servers can be used when needed depending on the type of Flent [57] test. Flent is an interesting network benchmarking tool that provides an evaluation of networks more reliably and swiftly. It is a wrapper for well-known network benchmarking tools such as NetPerf and Iperf [57]. It aggregates tests data series and also collects metadata from both local and remote hosts and stores it along with the plot data. This helps the reproducibility and authenticity of the tests performed.

## 5 Results and discussion

This section will provide the results of thorough tests performed on our physical testbed. It is divided into four sub-sections.

First, we have the **TCP Upload Test with 1/4/8/12/16 streams** in upload. The plots provided for this test are the *box plot of totals*, the *ping cumulative distribution (CDF) plot*, and the *queue backlog plots*. Secondly, we will provide results of a more rigorous form of network test known as **Realtime Response Under Load (RRUL).** This test is specially designed by the bufferbloat [58] community to stress-test networks. It consists of four streams in upload and four in download direction. It measures latency using both UDP and ICMP packets. The basic objective behind this test is to fully saturate the link and measure the most important performance metrics i.e., the throughput and the delay incurred. The same four plots are provided for this test. In this category, we choose **RRUL_BE** which is the RRUL test with best effort. We also conducted an **RTT fairness test** using two local Linux servers and two remote servers located in North America. For the sake of brevity tests with Wireless AC are presented in the paper and Wireless N results are available in our online data source [17]. Finally, the **Web (HTTP Delay Test)** has been performed to test the performance of AQMs for website fetch delay. In this case, rigorous testing has been performed with RRUL flow and a TCP flow started as cross traffic in the background.

### 5.1 TCP upload tests

The TCP upload tests, test the congestion controls and AQMs by sending data upstream. These tests are important as not only do they bring a vivid picture of CCA’s work, but also shed light on the AQM behavior which has been configured at the bottleneck. We start with a single stream test and gradually increase the number of streams to 4/8/12/16. We will see that these tests will prove helpful in evaluating our configured AQMs with BBR-n in our pursuit to find the ideal TCP-AQM pair.

#### 5.1.1 Single TCP upload test

**Fig 6:**
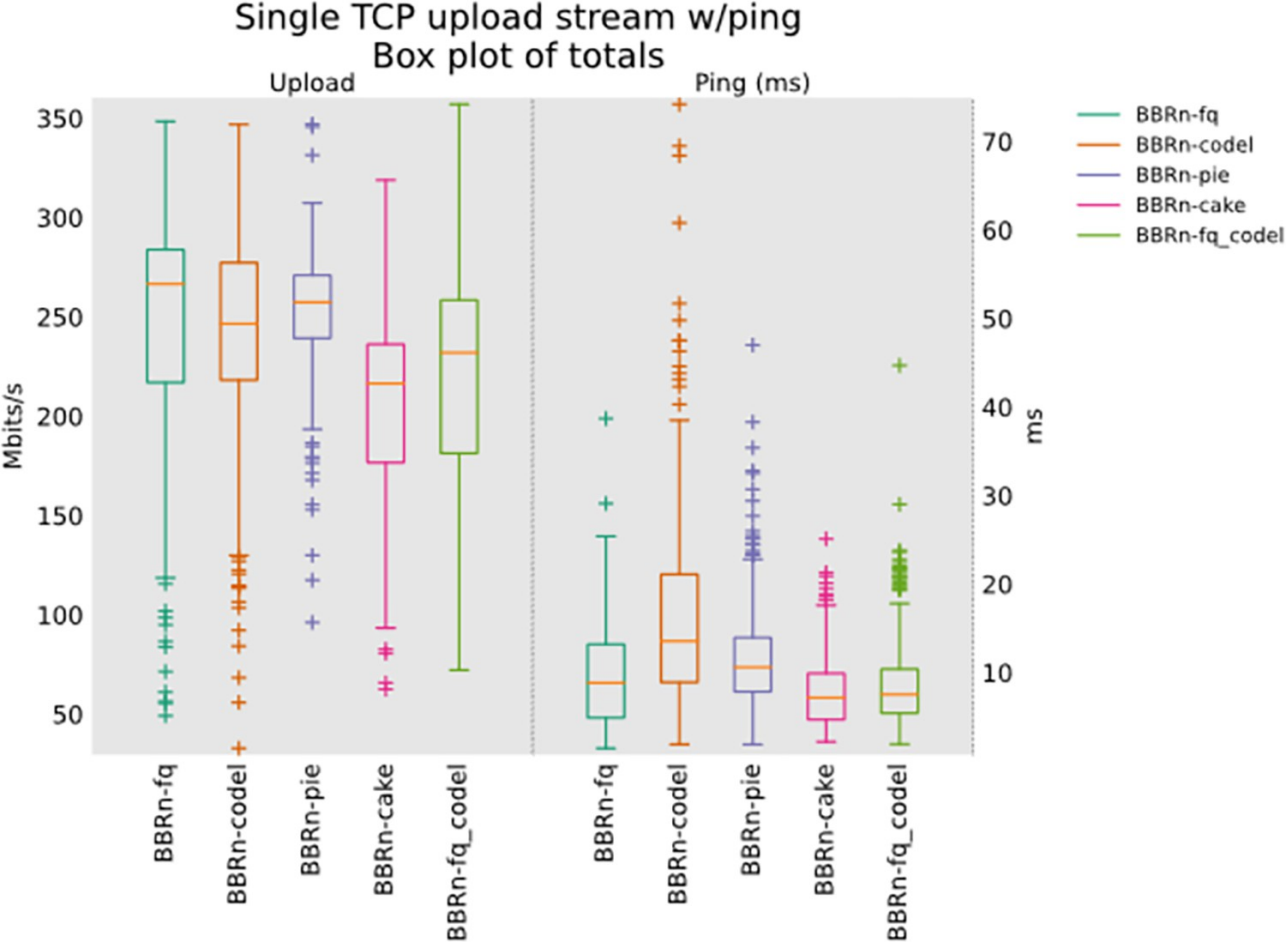
BBR-n with AQMs in single TCP upload test.

This test was performed with our proposed BBR-n as TCP congestion control. In this single stream test the whole available bandwidth of almost 275 Mbps was made available to a single flow for every AQM turn by turn. From **“Fig 6”**, we see that BBR-n provides the best throughput with FQ, but the lowest latency was observed with CAKE.

**Fig 7:**
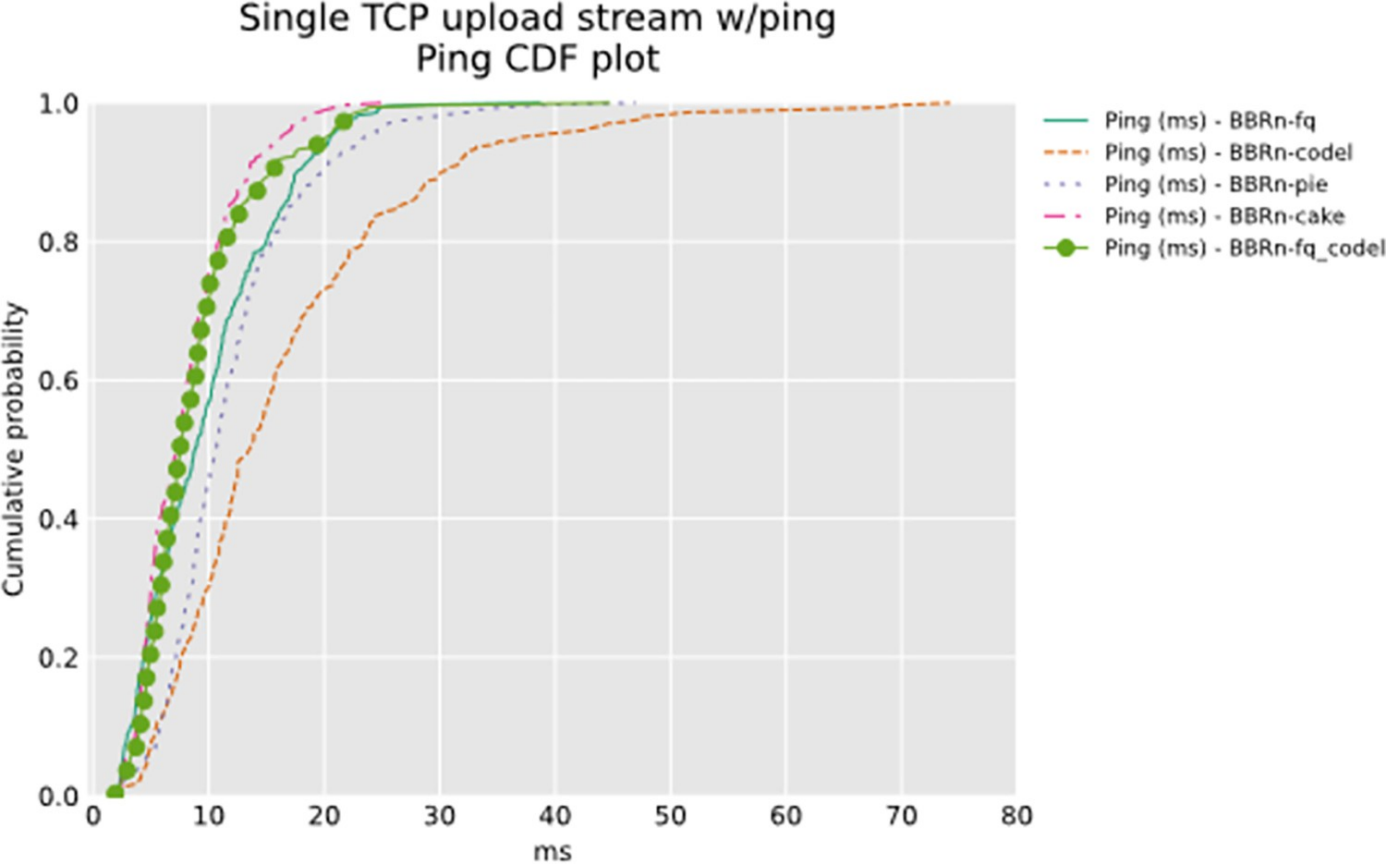
BBR-n with AQMs (ping CDF plot).

The Ping CDF plot in **“Fig 7”** shows that BBRn deployed with CAKE gives the lowest latency for a single TCP upload test done via the wireless bottleneck.

**Fig 8:**
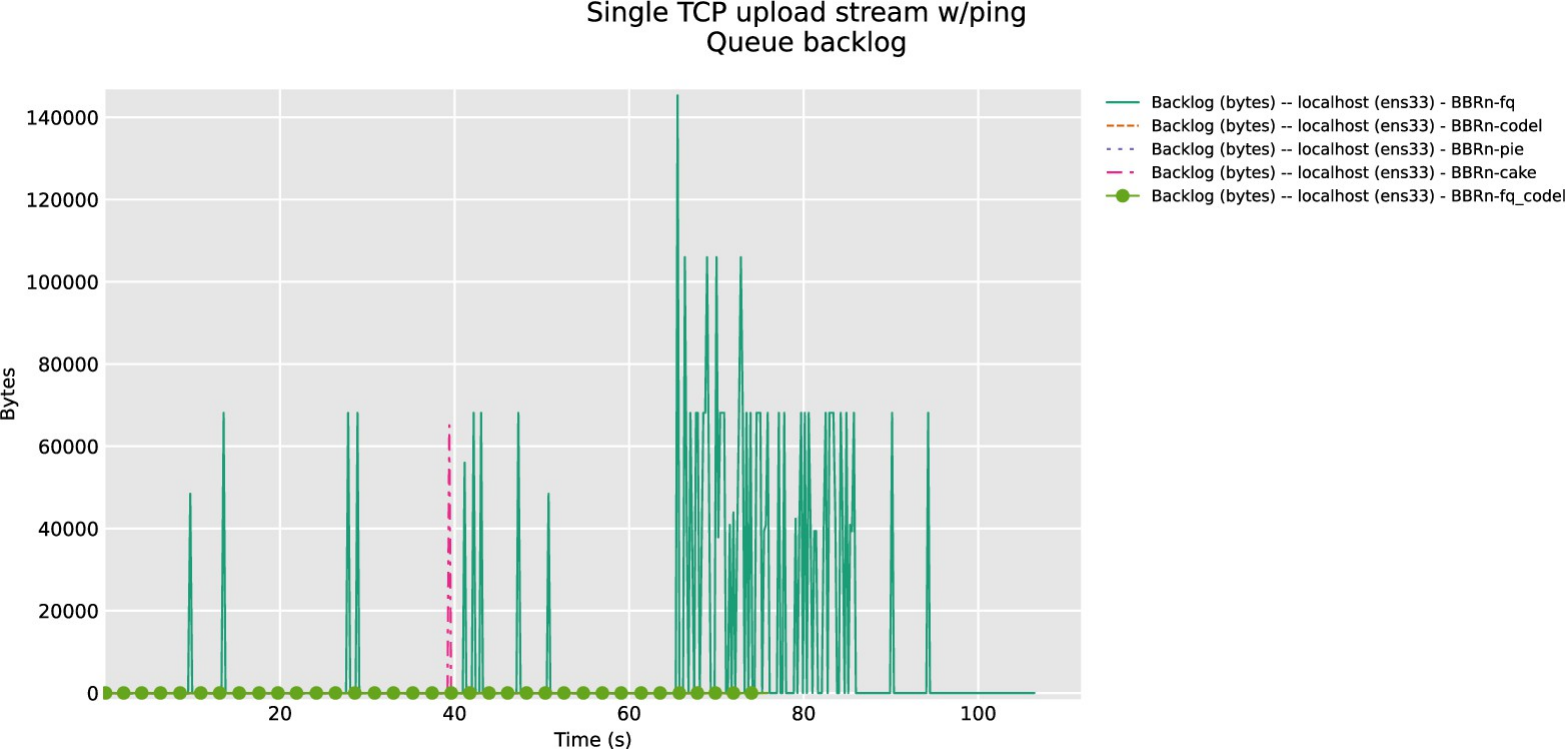
BBR-n with AQMs (queue backlog plot).

An interesting result is deduced from **“Fig 8”** in which we see that FQ is showing backlog bytes. With no backlog in the case of FQ_CoDel and PIE. Cake AQM does show a momentary pink spike with few backlog bytes which is not a big deal.

#### 5.1.2 Four TCP upload test

Next is our Four TCP stream test in Upload, the reason being to analyze the BBR-n and AQM performance with an increased number of flows in the uplink.

**Fig 9:**
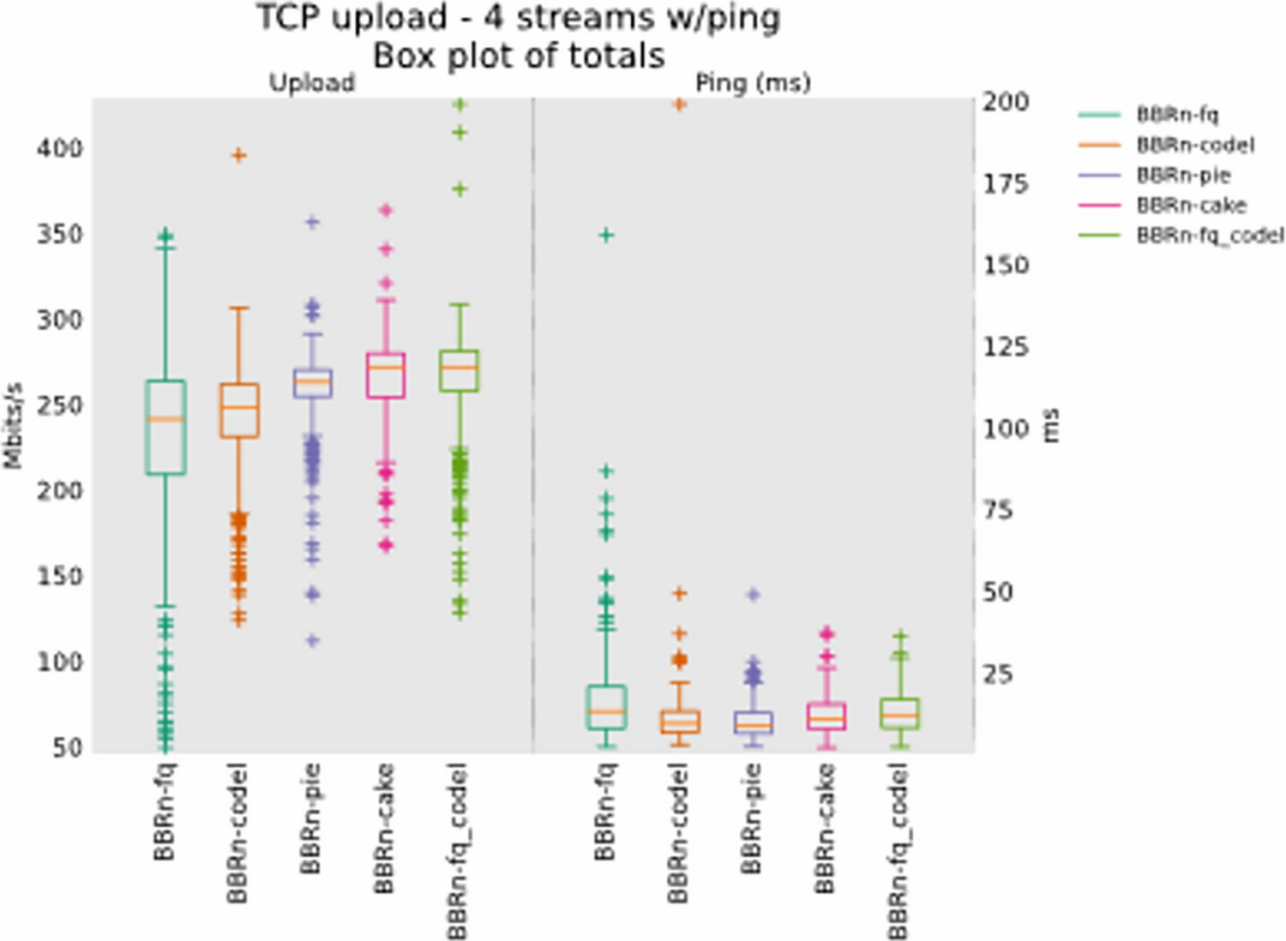
BBR-n with AQMs in four TCP upload streams test.

From **“Fig 9”**, we see that BBR-n provides better throughput with CAKE. The latency observed is also on a lower trend with CAKE. We can observe as the number of concurrent upload streams has increased; BBR-n performs better with CAKE in this test.

**Fig 10:**
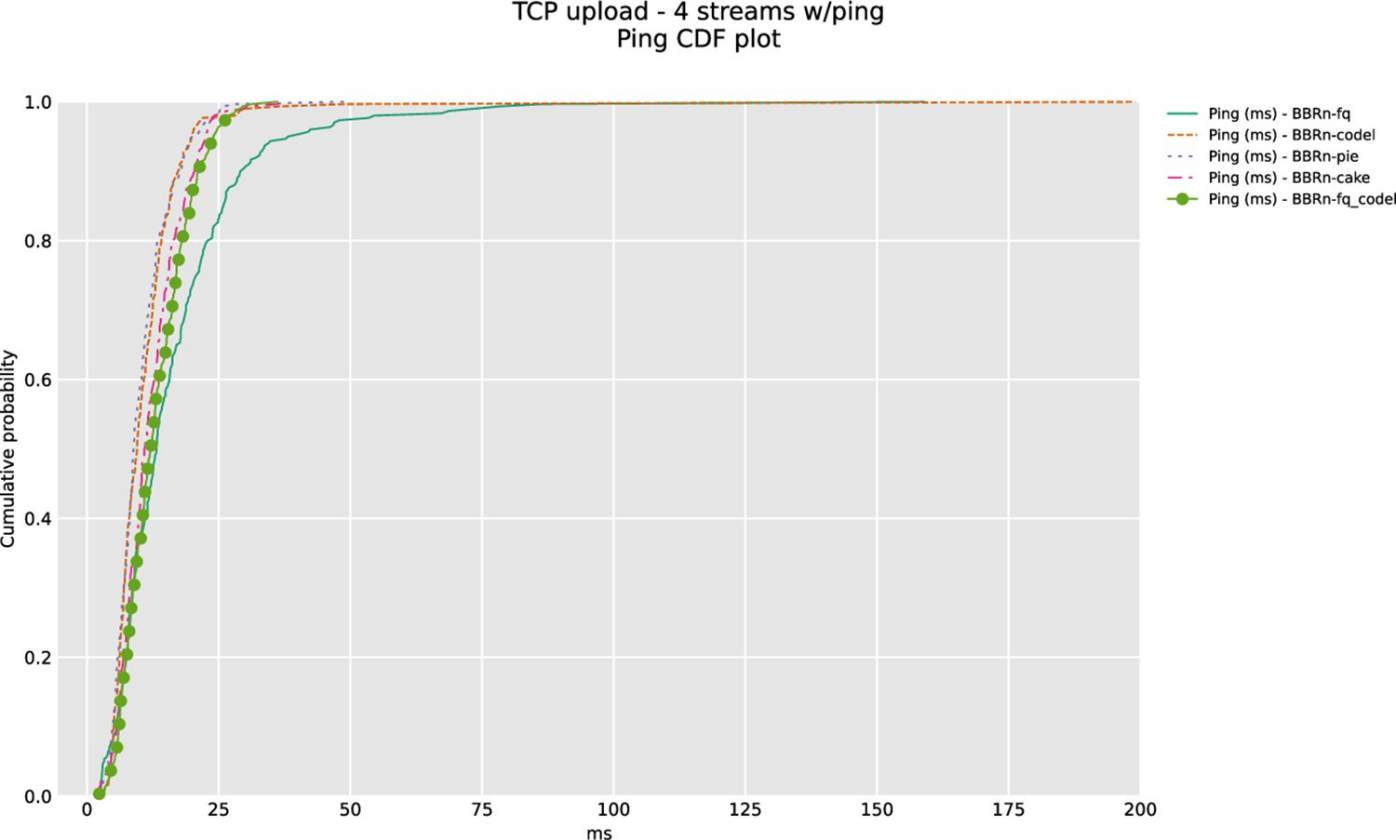
BBR-n with AQMs (ping CDF plot for four TCP streams).

The Ping CDF plot of **“Fig 10”** confirms that BBRn deployed with CAKE and PIE here gives the lowest latency.

**Fig 11:**
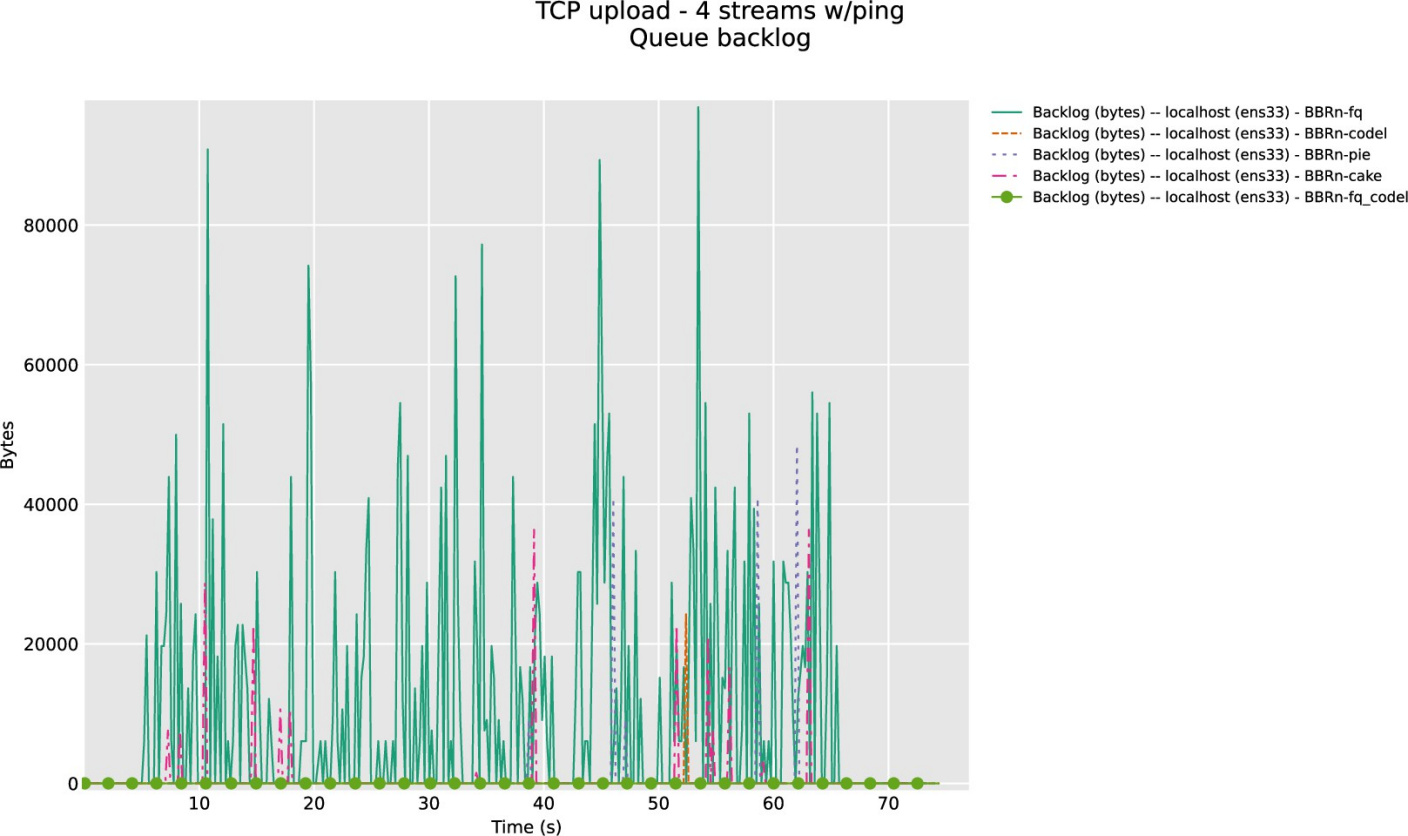
BBR-n with AQMS (queue backlog with four TCP streams).

From **“Fig 11”** we see that FQ is giving a lot of backlog bytes. So, we need to have a different AQM that can work with BBR-n giving an optimum throughput and low latency that is a crucial requirement for all Wi-Fi. By default, BBRgen works with FQ, but our tests show very clearly that due to the pacing mismatch between the BBRgen internal pacing and the pacing deployed by FQ results in backlog bytes.

#### 5.1.3 Eight TCP upload test

**Fig 12:**
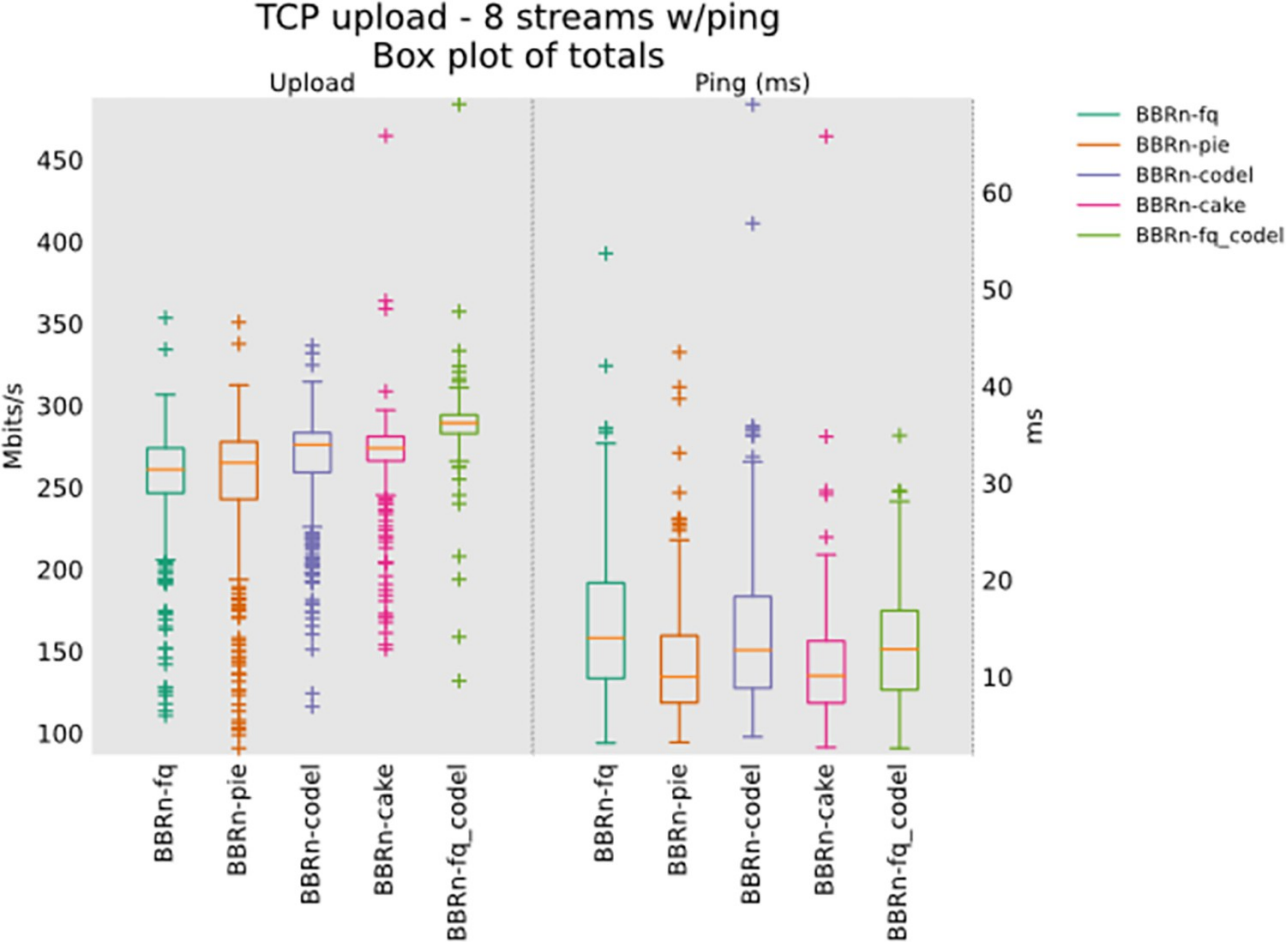
BBR-n with AQMs in eight TCP upload streams test.

We gradually increased the flows to eight now in the uplink direction. From **“Fig 12”** we see that BBR-n provides a good throughput with CAKE. The latency observed is also the lowest for CAKE, but we will see further progress in upcoming tests about the queue backlog bytes for CAKE.

**Fig 13:**
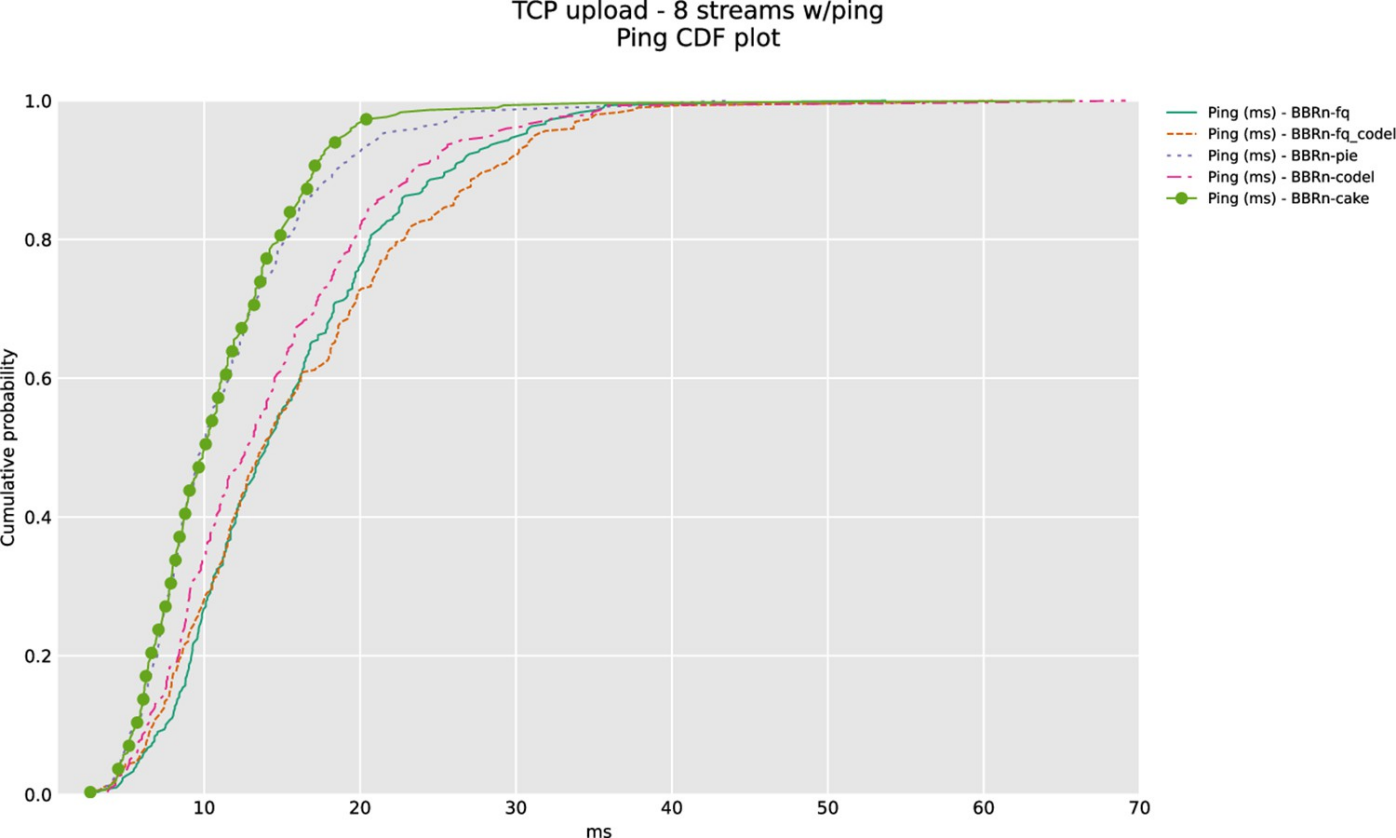
BBR-n with AQMs (ping CDF plot for eight TCP streams).

CAKE leads with the lowest latency in Ping CDF as depicted in “**Fig 13”**.

**Fig 14:**
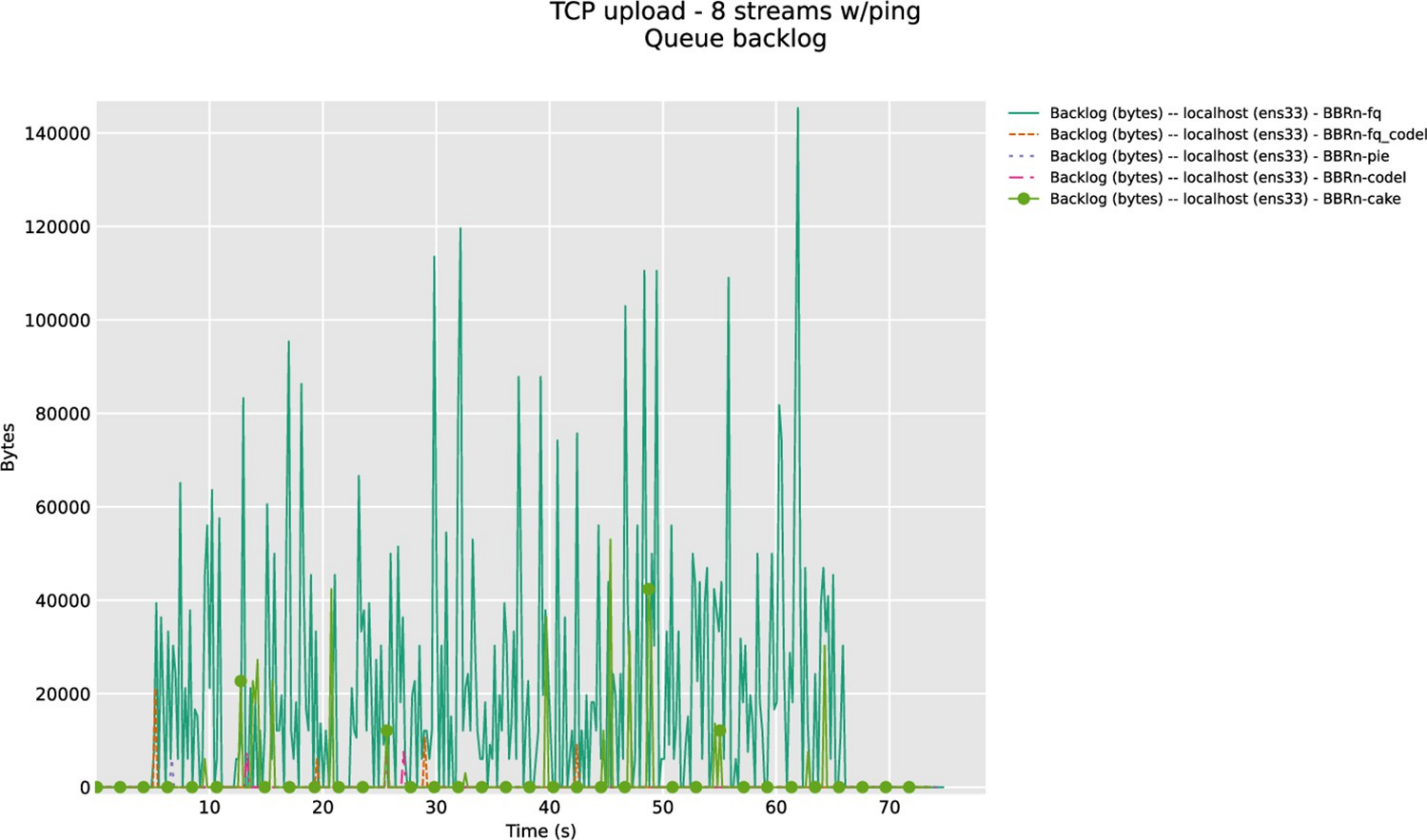
BBR-n with AQMs (queue backlog with Eight TCP streams).

CoDel and CAKE have a negligible backlog in this test done with 8 streams in upload as shown in **“Fig 14”**.

#### 5.1.4 Twelve TCP upload test

**Fig 15:**
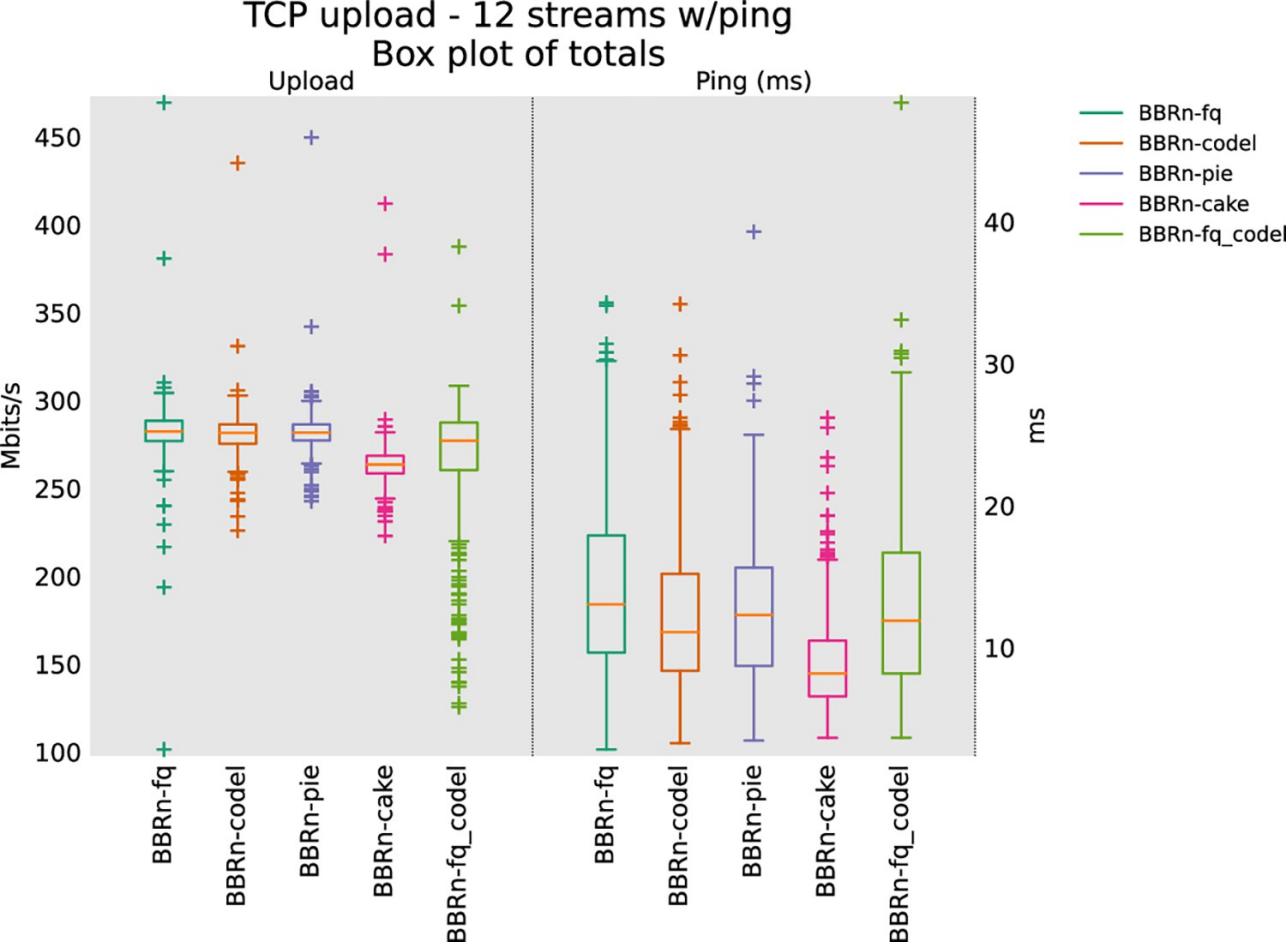
BBR-n with AQMs in twelve TCP upload test.

**Fig 16:**
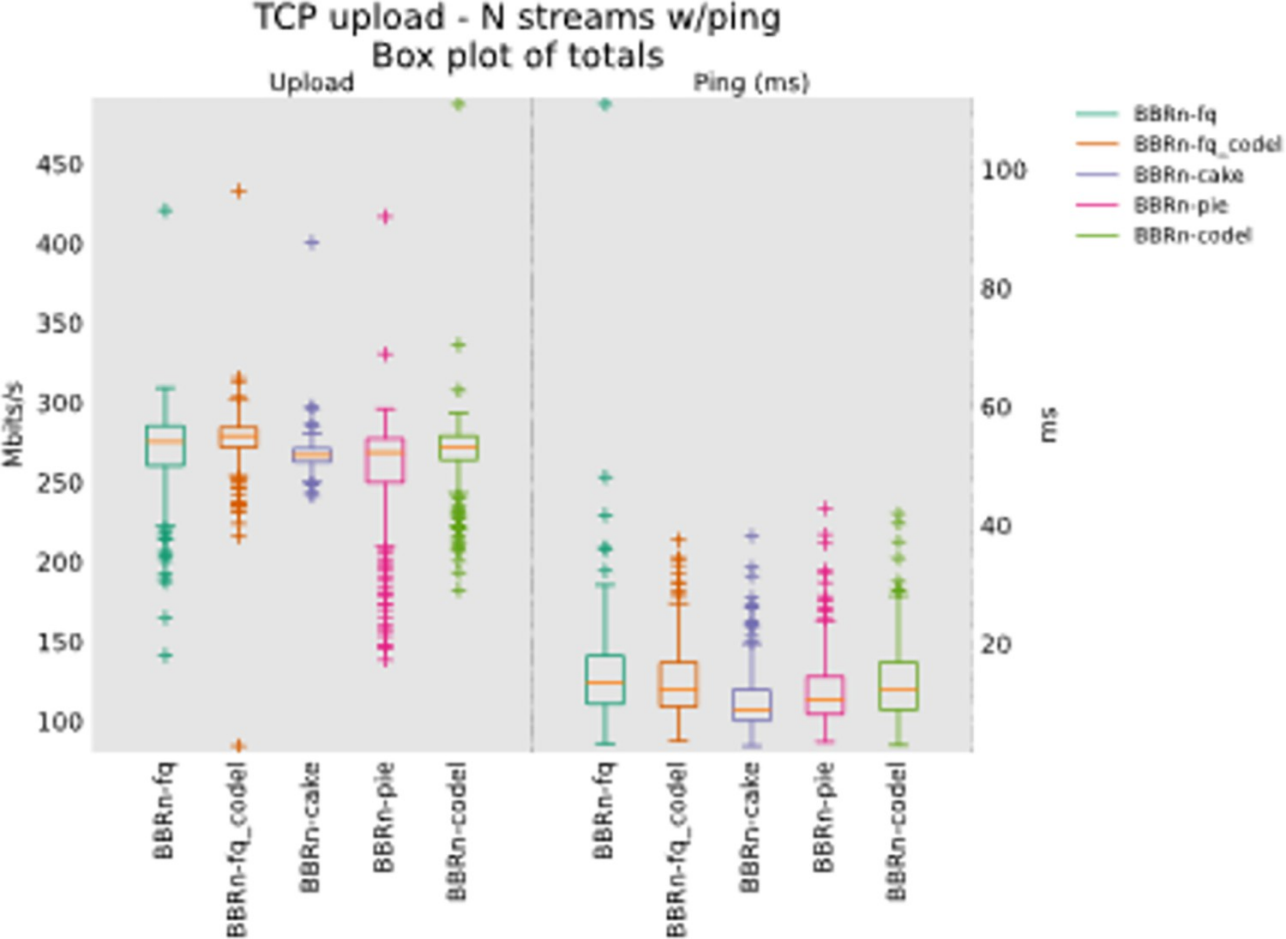
BBR-n with AQMs in sixteen TCP upload test.

From the 12 and 16 streams upload test results as shown in **“Figs 15 and 16”**, it is clear that CAKE provides the lowest latency. As far as throughput is concerned it is neck on neck with FQ_CoDel. These upload tests conclude that BBR-n along with CAKE is the best TCP-AQM pair to be deployed in TCP uplink scenarios with Wi-Fi 5 when we have more streams in Upload, which is typically the case of a normal user using Wi-Fi with many streams in Uplink and Downlink directions.

In **“Table 4”** five CCAs of “**Fig 16”** are analyzed via the Analysis of Variance (ANOVA) [59] test for their Ping latencies.

**Table 4:** ANOVA results for ping for BBR-n with modern AQMs.

Anova: Single Factor						
		
SUMMARY						
Groups	Count	Sum	Average	Variance		
Ping (ms) ICMP—BBRn-fq	350	4602.022074	13.1486345	67.84509788		
Ping (ms) ICMP—BBRn-fq_codel	350	4243.574833	12.12449952	42.19142717		
Ping (ms) ICMP—BBRn-cake	350	3243.585484	9.267387097	25.4507227		
Ping (ms) ICMP—BBRn-pie	350	3785.338317	10.81525234	36.46676389		
Ping (ms) ICMP—BBRn-codel	350	4205.858819	12.01673948	46.25007793		
ANOVA						
Source of Variation	SS	df	MS	F	P-value	F crit
Between Groups	3088.821469	4	772.2053672	17.69456679	3.00E-14	2.377027281
Within Groups	76153.22726	1745	43.64081791			
						
Total	79242.04873	1749				

For comparison of a data set comprising more than two groups, ANOVA single factor is the right choice instead of the T-test. As we have multiple groups of data collected for throughput and latency for different AQMs, we opted for this test. For this test to run on a data set, a significance level “α” is assumed. We opted for the default “α” value of 0.05. At the top of the table, we have the summary of the sums, averages, and variances of the groups being compared using 350 data points for each of the groups. At the bottom of the table, we have the ANOVA results. The “SS” column represents the sum of the squares between and within groups. “df” represents the degrees of freedom and “MS” the mean squared values. The ratio of MS for both “between groups and within groups” is known to follow the F distribution. Therefore, to get a statistical conclusion we compare this F value calculated from the data set with the F critical value (F crit) at a significance level “α” of 0.05 in the F table. Since this value of F of 17.69 is greater than the F crit value of 2.37, the results in **“Table 4”** may be interpreted as statistically significant among the means of the group at the significance level “α” of 0.05. The P-value of 3.00E-14 is well below the threshold value of 0.05 used in the ANOVA test proving that our results are statistically significant [60].

The ANOVA results for the analysis performed on the Ping latency data set are shown in **“Table 4”**. We see that BBR-n Ping latency with CAKE AQM is 9.2 ms, which is lower than what we are getting with other AQMs. i.e. 13.14 ms with FQ, 2.12 with FQ_CoDEL, 10.81 ms with PIE, and 12.01 ms with CODEL.

### 5.2 RRUL_BE (Realtime Response Under Load Exclusively Best Effort) test

**Fig 17:**
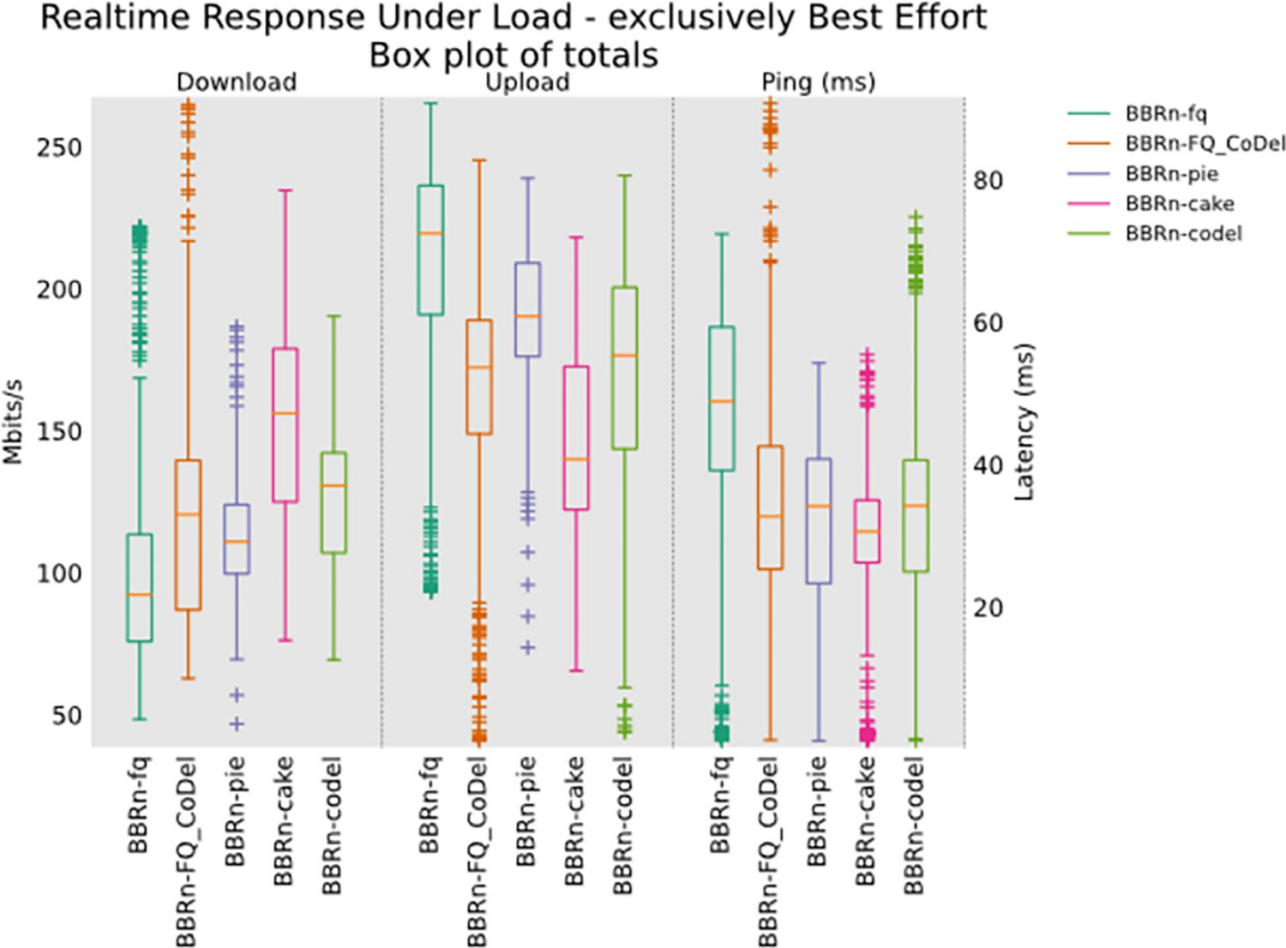
RRUL best effort test.

Next, is the RRUL_BE test, which is an integrated test in Flent. It uses eight TCP streams. Four in upload and four in download. This test has been created with one main purpose in mind, which is to stress the network as much as possible while keeping fairness. It is the same test as RRUL without classification. It is claimed to be the fairest test of all by the bufferbloat community. The results from this test are shown in “**Fig 17”**.

The CAKE leads by providing fairness in both uplink and downlink paths as is evident from box totals plots of **“Fig 17”**. The other AQMs as seen are rather unfair and provide more variations in throughput in uplink/downlink. The latency of CAKE is lowest as shown in **“Fig 17”**.

The ANOVA results for Ping data gathered from the RRUL_BE test are shown in **“Table 5”**. The Ping latency for BBR-n with CAKE is the lowest at 24.5 ms and BBR-n with FQ has the highest latency of 45.09 ms. This test data set was also 350 data points taken at a step size of 0.2 s for 60 s for each of the AQM. From the ANOVA table, we get an F value of 39.6, which is greater than the F crit value of 2.37, and a P-value of 8.56E-32 which is far below the threshold values of 0.05 showing that our results are statistically significant.

**Table 5:** ANOVA results for ping for BBR-n with other AQMs.

Anova: Single Factor						
SUMMARY						
*Groups*	*Count*	*Sum*	*Average*	*Variance*		
Ping (ms) ICMP—BBRn-fq	351	15827.63	45.09297	590.2601		
Ping (ms) ICMP—BBRn-FQ_CoDel	351	12149.71	34.61457	1285.412		
Ping (ms) ICMP—BBRn-pie	350	10011.27	28.60363	258.3802		
Ping (ms) ICMP—BBRn-cake	351	8601.419	24.50547	202.2645		
Ping (ms) ICMP—BBRn-codel	351	10117.8	28.82563	492.5026		
ANOVA						
*Source of Variation*	*SS*	*df*	*MS*	*F*	*P-value*	*F crit*
Between Groups	89667.46	4	22416.86	39.60999	8.56E-32	2.377016
Within Groups	989828.6	1749	565.9397			
Total	1079496	1753				

### 5.3 RTT fairness test

**Fig 18:**
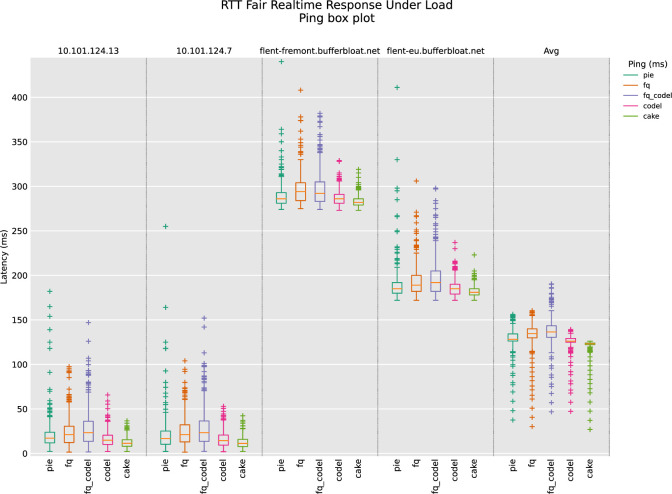
RTT fairness test (ping box plot).

RTT_Fair tests TCP performance between two or more hosts to see if a system is RTT-fair (meaning that connections machines at different distances eventually or not get a fair share of the bandwidth). For this test, we arranged two server Linux machines, one running kernel 6.2.0 and the other on 6.1.0. Their IP addresses are shown in **“Fig 18”**. The remaining two remote servers were selected by Flent tool itself as flent-fremont.bufferbloat.net and flent-eu.bufferbloat.net. The idea was to have two local and two remote servers at long distances to check the RTT fairness of AQMs. From the Ping box plot and the Ping CDF plot, it is clear that CAKE deployed with BBR-n is performing better. On local servers, the cake gives lower latency and on the remote servers, the cake is leading with the lowest latency.

ANOVA statistical analysis on the data set of **“Fig 18”** is given in **“Table 6”**. BBR-n with CAKE gives the lowest latency of 121.45 ms and the highest latency of 137.19 ms was observed with FQ_CoDEL. The statistical significance of our results is conformed from the F values of the ANOVA results which is 47.8 and is greater than the F crit value of 2.37. The P-value of 2.93E-38 is also way less than the threshold value of 0.05.

**Table 6:** ANOVA results for ping for BBR-n with other AQMs.

Anova: Single Factor						
SUMMARY						
*Groups*	*Count*	*Sum*	*Average*	*Variance*		
Ping (ms) avg—BBRn-cake	352	42753.71	121.4594	178.0266		
Ping (ms) avg—BBRn-codel	352	44253.82	125.7211	108.0851		
Ping (ms) avg—BBRn-fq_codel	352	48293.56	137.1976	434.1997		
Ping (ms) avg—BBRn-fq	351	46766.04	133.2366	332.3399		
Ping (ms) avg—BBRn-pie	352	45576.74	129.4794	344.8433		
ANOVA						
*Source of Variation*	*SS*	*df*	*MS*	*F*	*P-value*	*F crit*
Between Groups	53529.69	4	13382.42	47.88521	2.93E-38	2.377001
Within Groups	490188.3	1754	279.4688			
Total	543718	1758				

**Fig 19:**
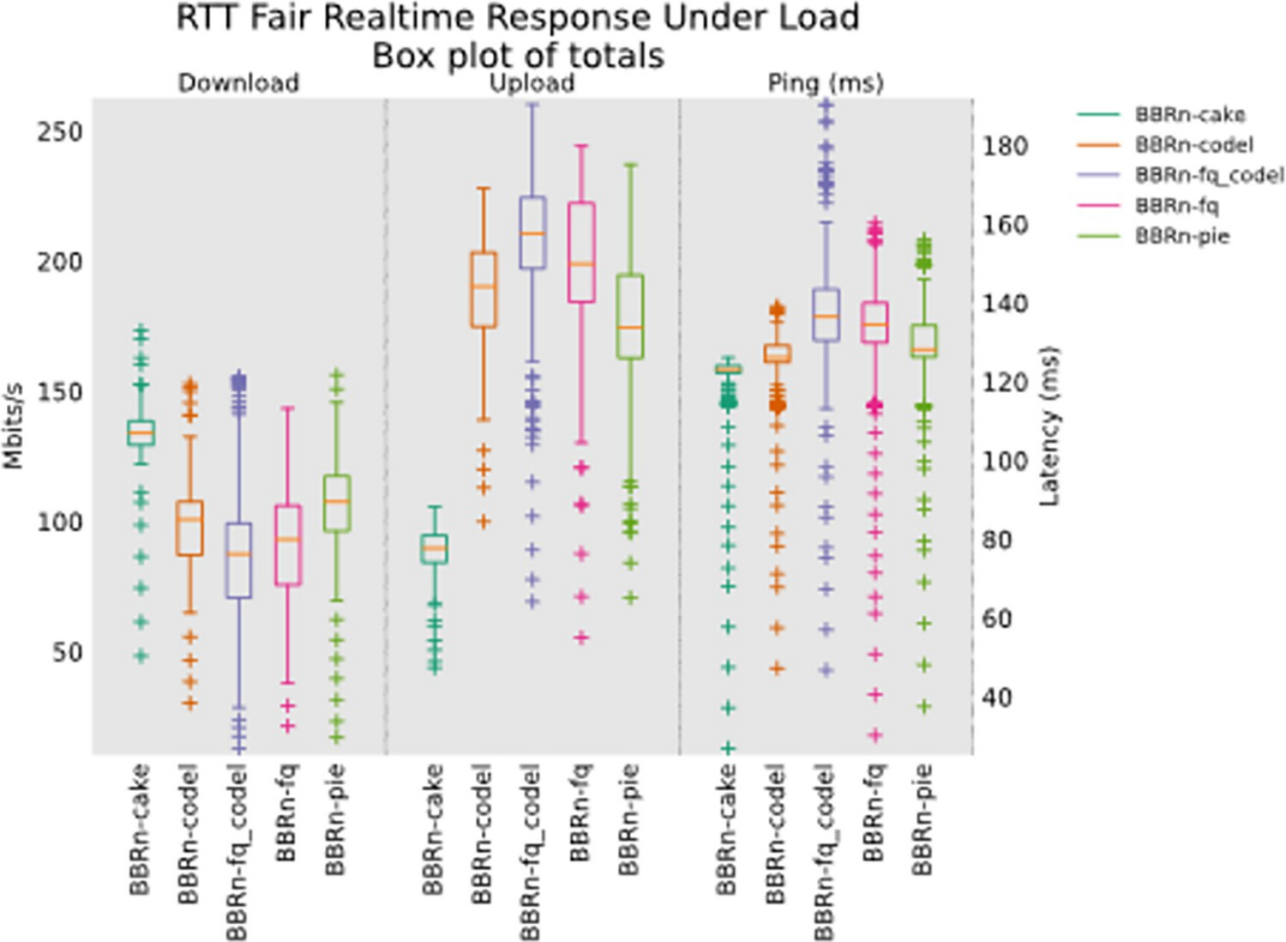
RTT fairness, RRUL test.

As seen from the box plot of totals RTT fair test for real-time response under load scenario in **“Fig 19”**, CAKE is performing balanced in both download and upload links along with the least latency of all AQMs. BBR-n-CAKE is indeed the Golden Pair that we were looking for and from the thorough and detailed tests under varying load conditions we have successfully proved it as well. For further confirmation, we have conducted an HTTP delay test in the next sub-section.

### 5.4 Web (HTTP delay) test

The purpose of this test is to measure the delay incurred when a user is accessing a website through the bottleneck that has been configured with the respective AQM scheme. A typical web fetch begins with a user client first performing a DNS lookup for the site being accessed, then it tries to fetch the main page or the base HTML page for the website and finally, a concurrent retrieval of all the objects associated with the web page starts over several TCP connections.

We define a performance metric known as *HTTP latency*, as the total time to retrieve all objects associated with the site under test with respective AQM activated at the bottleneck. We used the well-documented and tested cURL library [61] for our test client, which is an HTTP getter script file [62] for fetching URLs with cURL. We used two websites for this test, Google and PCMAG. Google Sites with 4.3 MB in 21 requests and PCMAG with 24 MB in 50 requests. This will help us analyze the web fetch delays for small and relatively large amounts of website data using different AQMs at the bottleneck with our proposed BBR-n. The test sites are repeatedly fetched by their URLs. For thorough testing, we got the lists of the objects for both sites in a text file and later on fed the URLs in that text file to the HTTP getter script file through the Flent command line. The performance metric we measured is the HTTP delay in ms. We ran this test again with RRUL traffic in the background as well as with the TCP upload traffic. The latter test demonstrates how timely the HTTP requests are delivered and that the web browsing experience will suffer if they are not delivered timely.

**Fig 20:**
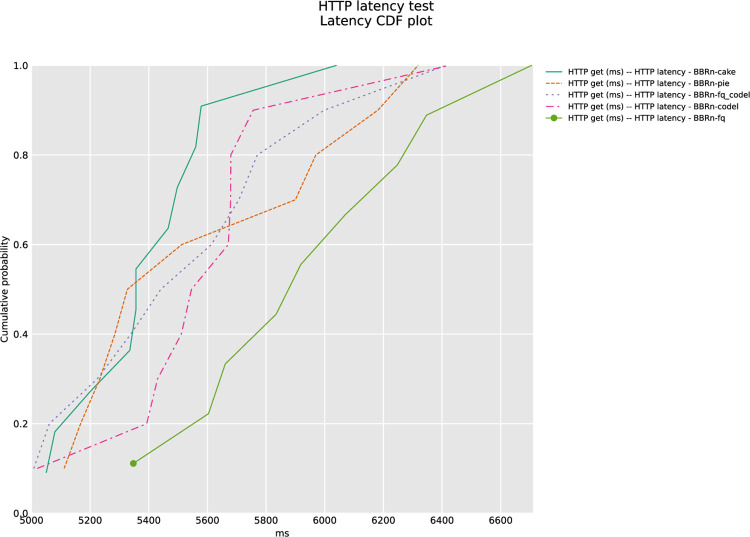
HTTP latency CDF plot for Google URLs.

**Fig 21:**
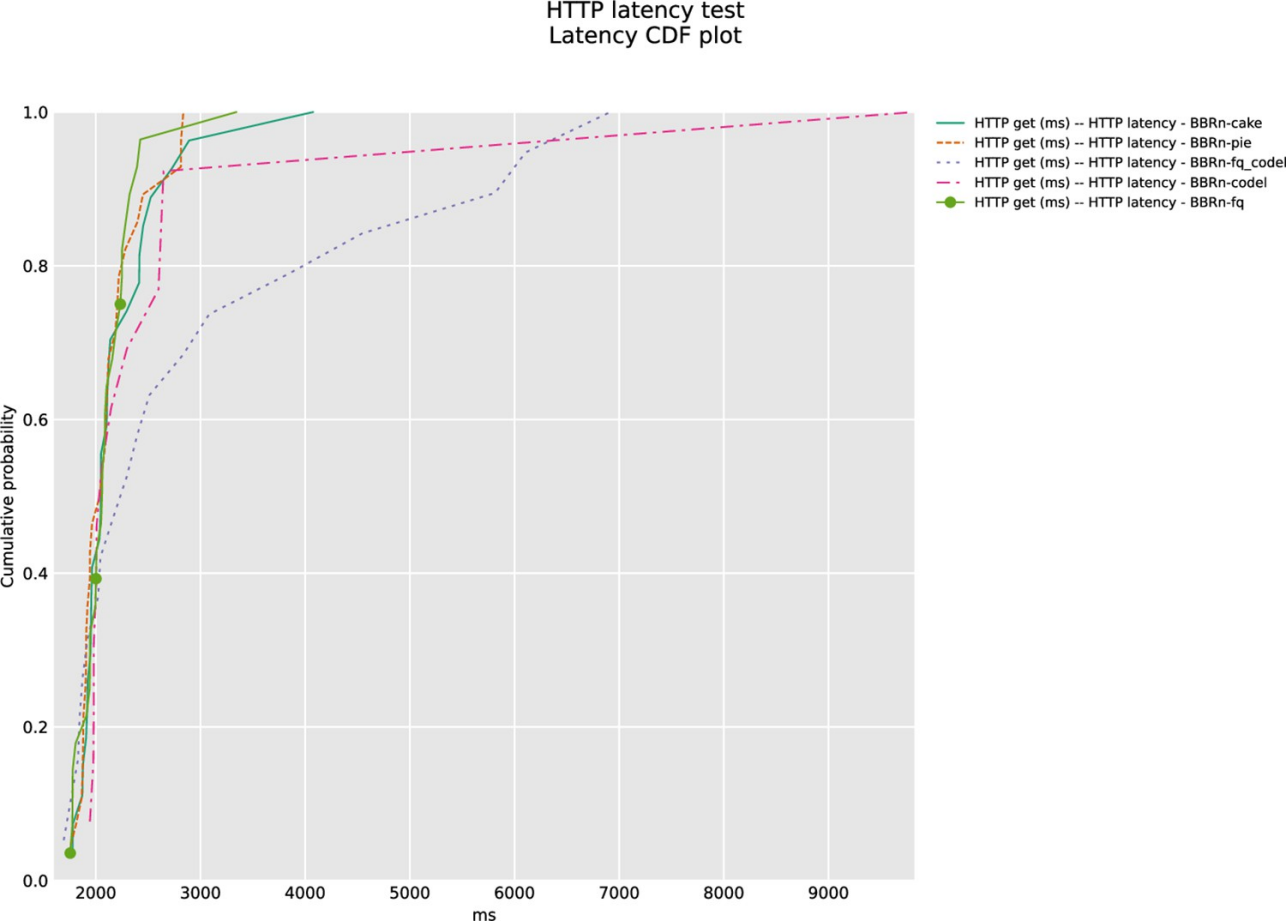
HTTP Latency CDF plot for PCMAG URLs.

The results of CDF plots are shown in **“Figs 20 and 21”**, confirming that CAKE is indeed performing best in this HTTP latency test.

The ANOVA statistical analysis was performed on the data set for **“Fig 20”**, the calculated HTTP latency averages are shown in **“Table 7”**. BBR-n with CAKE gives the lowest latency of 5409.41 ms. The F value is at 24.6 and is greater than the F crit value. The P-value of 4.2E-17 is well below the significance level of 0.05, which verifies our results are statistically significant.

**Table 7:** ANOVA results for HTTP latency for BBR-n with other AQMs.

Anova: Single Factor						
SUMMARY						
*Groups*	*Count*	*Sum*	*Average*	*Variance*		
HTTP latency—BBRn-fq	46	279221.9	6070.042	121486.6		
HTTP latency—BBRn-fq_codel	51	284309.5	5574.697	154506.5		
HTTP latency—BBRn-codel	49	276225.7	5637.258	100974.4		
HTTP latency—BBRn-pie	50	278430.5	5568.609	156273.1		
HTTP latency—BBRn-cake	51	275880.2	5409.416	60789.15		
ANOVA						
*Source of Variation*	*SS*	*df*	*MS*	*F*	*P-value*	*F crit*
Between Groups	11687423	4	2921856	24.60653	4.2E-17	2.408945
Within Groups	28735830	242	118743.1			
Total	40423253	246				

### 5.5 BBR-n with AQMs in a multi-node scenario

**Fig 22:**
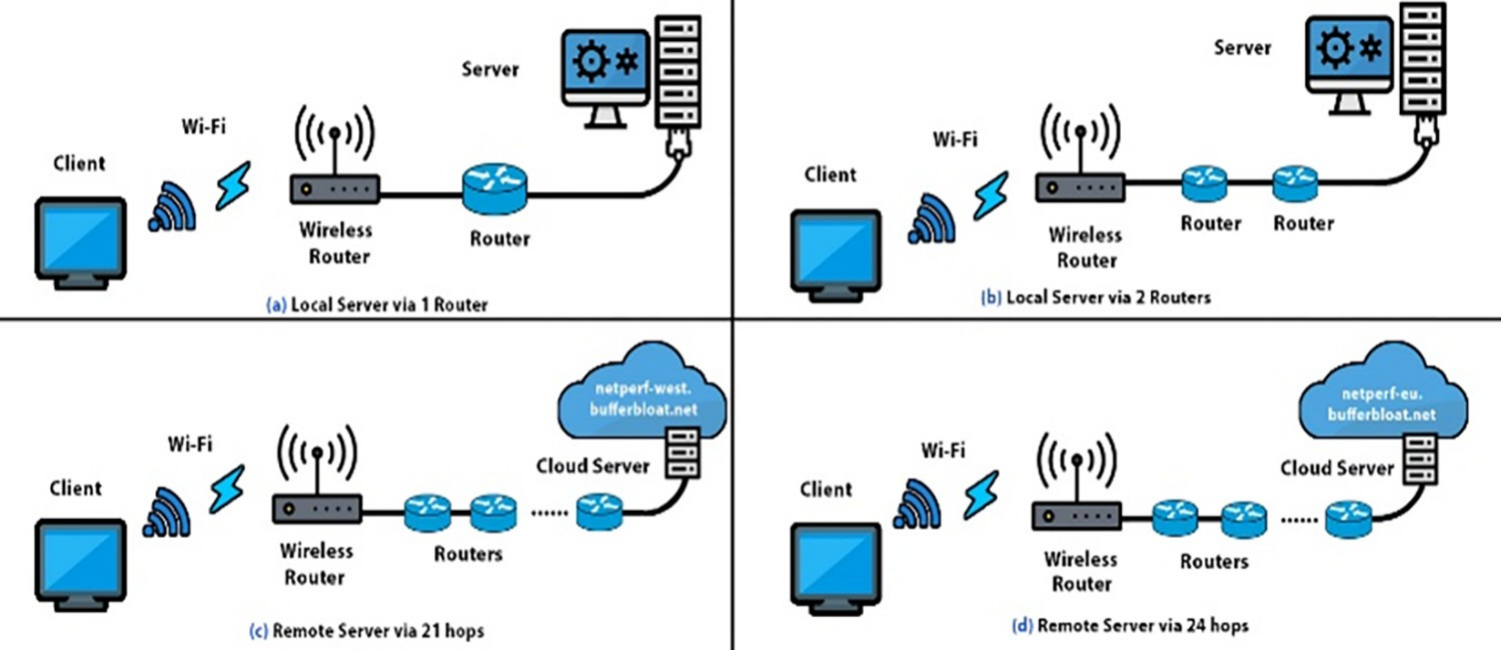
Physical testbed (multi-node). Shows a Linux-based wireless client connected to a Wi-Fi 4/5 router in a multi-node (local/remote) scenario to the Linux server. **Image source**: Physical testbed (multi-node), “Client, Wireless Router, Router, and Server icons” are imported from https://www.pngegg.com/ which is a free hosting site. CC BY 4.0.

**Fig 23:**
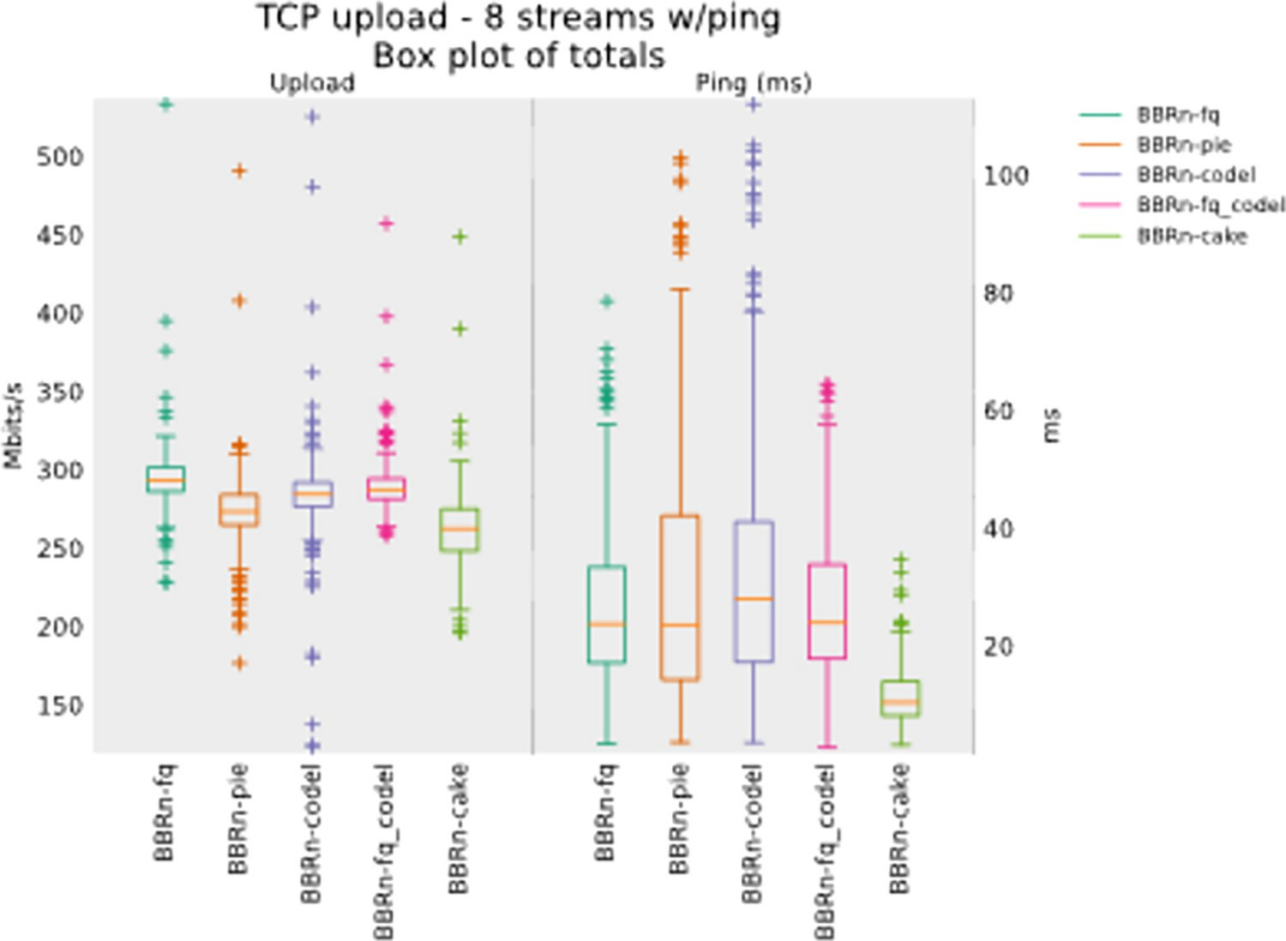
BBR-n tested with a single node addition.

**Fig 24:**
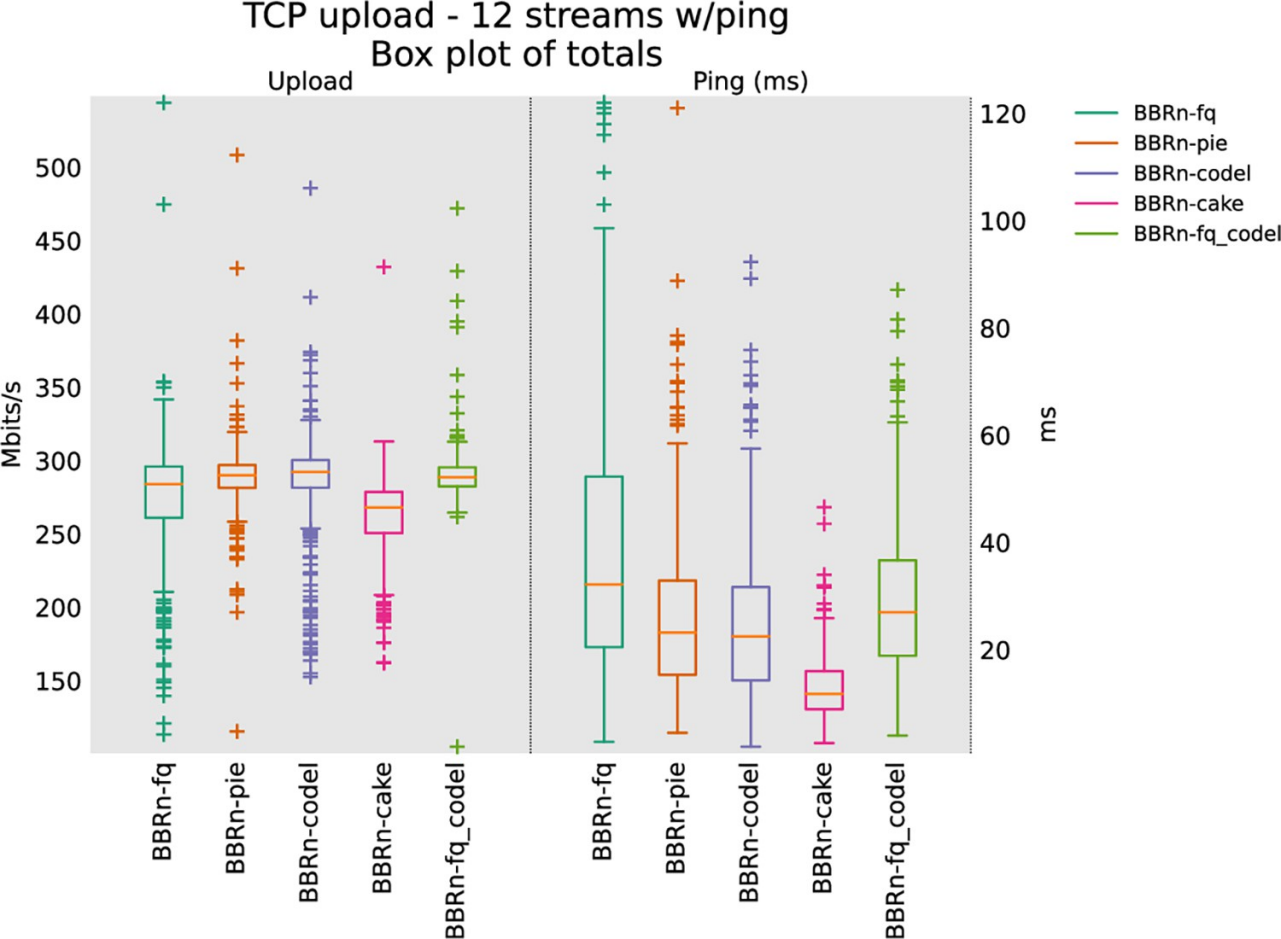
BBR-n tested with two nodes addition.

In this section, we modified our testbed of **“Fig 5”** by adding more local and remote nodes in our network path from the wireless bottleneck to the wired server. The modified diagram is shown in **“Fig 22”**, it consists of four parts, a, b, c, and d. At the top of our diagram, ‘a’ and ‘b’ show the local server connectivity with added nodes, whereas at the bottom of the diagram, ‘c’ and ‘d’ show the remote server connectivity with multiple hops involved. The objective was to analyze the effect of increased node density on the interplay of BBR-n with modern AQMs. 8/12 TCP Upload stream tests were performed in this case and the results are shared in **“Figs 23 and 24”**. From the results, we see that although more nodes were added to the network path which increased latency, CAKE still performed well. Its Ping latency is very low due to the refinements provided in its traffic shaper, flow isolation, AQM, and packet management.

Moreover, the results and statistical analysis from the tests done on two remote servers, flent-fremont.bufferbloat.net, and flent-eu.bufferbloat.net, which were 24 and 21 hops away from our client machine respectively, are shared in **“Fig 18”** and **“Table 6”.** CAKE AQM did very well here too. These results show that CAKE AQM configured at the wireless bottleneck leads to much lower delays than other AQMs, even with an increased node density. Both local and remote nodes were added to the network path and CAKE’s overall latency was better than the other AQMs that were tested.

## 6 Conclusion and future directions

In this paper, we have provided an in-depth analysis of the five most common AQMs available in Linux kernel 6.2.x.x with the latest TCP congestion control algorithm BBRs’ variant BBR-n. We used our proposed and tested BBR variant which we call BBR-n (BBR new) in our pursuit to find the ideal pair of AQM that suits best in a wireless scenario. In the 16 streams, the TCP Upload test CAKE comes up with a minimum latency of 9.2 ms. In the RRUL_BE test CAKE gives a min latency of 24.5 ms as compared with the 28 ms latency of FQ_CoDeL. It wins the race again in the RTT fairness test with the least latency of 121 ms. Finally, in the web fetch test, it comes out victorious with a least latency of 5.4 secs. Queue backlogs are also negligible in the case of CAKE. Various performance metrics such as throughput, latency, RTT variations as well as queue backlog, and the fairness between the flows using the RTT fair test at the wireless gateway were used to prove our claim of the best TCP-AQM pair HTTP delay test results show that BBR-n when deployed with CAKE AQM, gave the best possible results in the wireless networking scenario that was used to test it and is the “*Golden Pair”* that we were trying to find.

While the use of better queue management techniques is on the rise, deployment is a major challenge. Since the internet is a collection of heterogeneous networks, we can expect varying behaviour in it. It is hard to find a solution that fits all kinds of networking scenarios. The queue management role is very important in ensuring that tomorrow’s internet is free from latency and that the issue of bufferbloat is minimized as much as possible. It is encouraging that we have open source operating systems such as Linux, which is evolving rapidly, and researchers across the globe our giving their inputs to improve the queue management techniques and suggesting new hybrids from the existing solutions. CAKE, which we have analyzed is also a result of such a venture and we saw from our rigorous tests that it proved to be very effective with BBR-n in a Wi-Fi 4/5-based scenario. This work of ours is expected to bring a new motivation in the scientific community to keep the research going in this very important direction i.e. not only striving for a robust algorithm for the transport layer, but an optimum queueing algorithm as well for the network layer that can complement it fully. Wi-Fi 4/5 are the two commonly used Wi-Fi standards in end systems. The research presented in this paper with a variety of tests on a physical wireless testbed is expected to raise more interest with its testing in the Wi-Fi 6/7-based wireless bottlenecks. The cybersecurity-related aspects can surely be coupled with the TCP-AQM pair to further fine-tune any AQM. In this case, the approaches briefly discussed such as deep neural network-based botnet detection and classification, development of convolutional neural network-based network intrusion detection system for edge computing, and a deep neural network-based botnet prediction models can help further probe on identifying the malicious attackers from the real-time datasets gathered from any type of wireless networks.
